# Species-Specific Trait Responses of Three Tropical Seagrasses to Multiple Stressors: The Case of Increasing Temperature and Nutrient Enrichment

**DOI:** 10.3389/fpls.2020.571363

**Published:** 2020-11-05

**Authors:** Inés G. Viana, Agustín Moreira-Saporiti, Mirta Teichberg

**Affiliations:** ^1^Department of Ecology and Animal Biology, University of Vigo, Vigo, Spain; ^2^Leibniz Centre for Tropical Marine Research GmbH, Bremen, Germany; ^3^Faculty of Biology and Chemistry, University of Bremen, Bremen, Germany

**Keywords:** *Cymodocea serrulata*, *Thalassia hemprichii*, *Halophila stipulacea*, morphology, storage, photophysiology, nutrient allocation, interactive effect

## Abstract

Seagrass meadows are declining globally. The decrease of seagrass area is influenced by the simultaneous occurrence of many factors at the local and global scale, including nutrient enrichment and climate change. This study aims to find out how increasing temperature and nutrient enrichment affect the morphological, biochemical and physiological responses of three coexisting tropical species, *Thalassia hemprichii*, *Cymodocea serrulata* and *Halophila stipulacea*. To achieve these aims, a 1-month experiment under laboratory conditions combining two temperature (maximum ambient temperature and current average temperature) and two nutrient (high and low N and P concentrations) treatments was conducted. The results showed that the seagrasses were differentially affected by all treatments depending on their life-history strategies. Under higher temperature treatments, *C. serrulata* showed photo-acclimation strategies, while *T. hemprichii* showed decreased photo-physiological performance. In contrast, *T. hemprichii* was resistant to nutrient over-enrichment, showing enhanced nutrient content and physiological changes, but *C. serrulata* suffered BG nutrient loss. The limited response of *H. stipulacea* to nutrient enrichment or high temperature suggests that this seagrass is a tolerant species that may have a dormancy state with lower photosynthetic performance and smaller-size individuals. Interaction between both factors was limited and generally showed antagonistic effects only on morphological and biochemical traits, but not on physiological traits. These results highlight the different effects and strategies co-inhabiting seagrasses have in response to environmental changes, showing winners and losers of a climate change scenario that may eventually cause biodiversity loss. Trait responses to these stressors could potentially make the seagrasses weaker to cope with following events, due to BG biomass or nutrient loss. This is of importance as biodiversity loss in tropical seagrass ecosystems could change the overall effectiveness of ecosystem functions and services provided by the seagrass meadows.

## Introduction

Seagrass meadows are valued for the ecosystem services they provide that have been recorded in an increasing number of studies (Costanza et al., 1997; Kirsch et al., 2002; Romero et al., 2006; Fourqurean et al., 2012; Ondiviela et al., 2014; Dewsbury et al., 2016; Nordlund et al., 2016). Despite their global significance and them being threatened by multiple anthropogenic stressors that affect their biodiversity and functioning (Lu et al., 2018) the attention paid to seagrass meadows is much lower than other coastal ecosystems, such as coral reefs (Unsworth et al., 2019).

The most prominent stressor of seagrasses generated locally is cultural eutrophication caused by increased loading of nutrients from human activities (Beiras, 2018). During the last decades, losses in seagrass meadows have been documented worldwide, especially in quiet, and poorly flushed estuaries where nutrient loads are intense and frequent (Burkholder et al., 2007). Contrary to temperate ecosystems, in the oligotrophic tropical environments, seagrass productivity is mainly limited by nutrients and not by light irradiance (Short, 1987); therefore, nutrient inputs could result in drastic changes in diversity loss (Kamermans et al., 2002). As seagrasses are the only submerged marine angiosperms, they can access both nutrients from water column and pore water. Thus, below-ground (BG) tissues play a key role in taking up different nutrient sources to meet *in situ* demands and translocating them along the plant (Viana et al., 2019a). This is especially important in the tropics as nitrogen (N) is constantly available in the pore water at higher concentrations than in the water column, and leaf turnover is constant throughout the year, whereas BG tissues have lower turnover rates (Duarte, 1991). Seagrasses are able to take advantage of nutrient pulses by increasing the enzymatic activity in response to uptake and assimilation and storing the incorporated nutrients (Viana et al., 2019a). In this way, nutrient additions can result in higher growth rates, primary productivity or nutrient content (Duarte, 1990; Agawin et al., 1996; Terrados et al., 1999; Ferdie and Fourqurean, 2004). However, if eutrophication persists, changes in biotic or abiotic interactions might occur, including algal blooms that increase nutrient competition and light deprivation, alterations in top-down regulation because of changes in leaf palatability, or increasing organic matter in the sediments that might create anoxic environments for benthic organisms (Burkholder et al., 2007). All these changes may ultimately affect species fitness and survival.

Although nutrient over-enrichment has been one of the main drivers of seagrass mortality worldwide, other abiotic factors, including temperature, play an important role in species distribution and survival. Temperature is one of the main drivers of biochemical reactions, significantly affecting growth, photosynthesis, sexual reproduction and survival (Durako and Moffler, 1985; Bulthuis, 1987; Lee et al., 2007; Xu et al., 2016; Zayas-Santiago et al., 2020). Temperature also affects seagrass nutrient content, as lower carbon (C) and N concentrations and higher C:N ratios have been observed at increasing temperatures (Kaldy, 2014; Mvungi and Pillay, 2019; Ontoria et al., 2019b). While moderate elevations in temperature might be positive for seagrass performance (Artika et al., 2020), the individual fitness is lost once the thermal optimum range is exceeded often leading to increasing mortality rates (Collier and Waycott, 2014). Tropical seagrasses usually grow at their upper optimal temperature limit, therefore changes in mean seawater temperatures might be critical (Koch et al., 2013). Changes in seagrass population and community structures have already been observed in areas affected by climate change (Jordà et al., 2012; Kendrick et al., 2019; Beca-Carretero et al., accepted) and will become more relevant under future climate change. Therefore, the knowledge accumulated on the effects of these individual stressors through field and laboratory experiments on temperate and tropical seagrasses is very extensive (see as example Bulthuis, 1987; Burkholder et al., 1994; Campbell et al., 2006; Winters et al., 2011; Collier and Waycott, 2014; Li et al., 2019).

In coastal systems, stressors rarely occur alone in the environment and, when acting together, their effects can be synergistic, additive or antagonistic (Todgham and Stillman, 2013; Gunderson et al., 2016; Stockbridge et al., 2020), although synergistic effects are most likely to occur when the stress events happen simultaneously or in quick succession (Gunderson et al., 2016). Accordingly, local anthropogenic impacts of human development, such as eutrophication, can combine with indirect consequences of climate change, including sea surface temperature or CO_2_ enrichment, causing even more dramatic consequences of future scenarios than initially predicted by single-factor experiments. As a result, the rates of change in seagrass ecosystems are faster than those experienced in their evolutionary history and may occur too fast to allow seagrasses to adapt (Orth et al., 2006; Waycott et al., 2009). Therefore, the interaction between stressors is now viewed as a critical issue, and it is suggested that single-factor experiments are not adequate for assessing the effects of several disturbances on coastal marine ecosystems (Wernberg et al., 2012; Todgham and Stillman, 2013; Ontoria et al., 2019b). In the last years, an increasing number of papers aiming to understand cumulative impacts of stressors have exponentially increased (Gunderson et al., 2016; Adams et al., 2020; Stockbridge et al., 2020), and more empirical data on the effects of the interaction of increasing temperature and nutrient over-enrichment at an individual level has been obtained (Touchette and Burkholder, 2002; Bintz et al., 2003; Touchette et al., 2003; Burnell et al., 2013; Kaldy, 2014; Kaldy et al., 2017; Egea et al., 2018; Moreno-Marín et al., 2018; Mvungi and Pillay, 2019; Ontoria et al., 2019b). Nevertheless, responses depend on their local adaptation and life history traits (Tuya et al., 2019; Anton et al., 2020) are species-specific, and to our knowledge, there is very limited information about the combined effects of these two stressors in any tropical seagrasses species (Artika et al., 2020).

The study of the response of seagrass individual traits, namely biochemical, morphological, and physiological traits, serve as early indicators of environmental change (Roca et al., 2016) before population level responses, such as changes in shoot density, biomass and species composition or biodiversity loss, are detected. This approach has been used in a wide number of studies to detect rapid responses (within weeks) to different stressors (Lee et al., 2007; Roca et al., 2016; Bertelli and Unsworth, 2018, and previously cited references). One important limitation of trait-based responses is that different stressors could cause the same effect, for instance, lower rhizome carbohydrate content is both observed after nutrient over-enrichment or reduced light exposures (Roca et al., 2016). More recent studies that simultaneously assess different plant individual traits have highlighted the importance of testing the responses at different levels of organization in the plants (Bertelli and Unsworth, 2018; Mvungi and Pillay, 2019). Moreover, a deeper understanding of the species-specific responses to stressors and their interaction is important as different combinations of seagrass traits may sustain different ecological functions that upscale to the ecosystem level (Barbier et al., 2011). Therefore, even though changes in seagrass traits could be seen as positive at an individual plant level (i.e., increasing photosynthetic rate) they could potentially change their related functions, negatively affecting the sustained ecosystem services (Burnell et al., 2013; Jiménez-Ramos et al., 2017; Soissons et al., 2018).

Tropical areas are seagrass biodiversity hotspots, gathering most of the 60 seagrass species that exist worldwide (Short et al., 2007). *Thalassia hemprichii* and *Cymodocea serrulata* are widely distributed in the Indo-Pacific bioregion. These two species have large blades and slow shoot turnover, especially *T. hemprichii* (Duarte, 1991). They both form persistent mixed or monospecific meadows that sustain food webs, including commercially important species (de la Torre-Castro et al., 2014). *Halophila stipulacea* is a tolerant species native to the Indo-Pacific bioregion that has colonized both the Mediterranean and Caribbean Seas (Winters et al., 2020). It has a smaller size than most seagrasses, so it frequently grows in sand patches or in the edges of bigger seagrass meadows, but it can also grow mixed with other macrophytes or form large monospecific meadows (Boudouresque et al., 2009; Sghaier et al., 2011). It presents faster shoot turnover and growth than the two other species (Duarte, 1991). All three species have been observed to tolerate different trophic conditions (Van Tussenbroek et al., 2016; Mwaura et al., 2017; Thomsen et al., 2020; Teichberg et al., *in preparation*) and adverse maximum temperatures (Campbell et al., 2006; Georgiou et al., 2016; Pedersen et al., 2016; Collier et al., 2017; George et al., 2018; Anton et al., 2020; Nguyen et al., 2020; Winters et al., 2020; Beca-Carretero et al., accepted). Furthermore, *T. hemprichii, C. serrulata* and *H. stipulacea* show differences in their life-history traits representing permanent, opportunistic and colonizing strategies, therefore different resistance and responses to stressors might be expected (O’Brien et al., 2018).

This study aims to find out how increasing temperature, nutrient over-enrichment, and the combination of both factors affect the morphological, biochemical and physiological responses of three common tropical Indo-Pacific seagrasses. To achieve this aim, combined temperature and nutrient enrichment laboratory experiments were conducted, and the responses in trait values of the three selected species were measured after 1 month under four different treatment combinations. We hypothesized that the combination of both factors will cause interactive (synergistic or antagonistic) responses in the three species, and these responses will be species-specific according to their life-history traits. The results of this study will provide important information on how the combined effects of climate change and nutrient enrichment will shape tropical seagrass meadows and their responses.

## Materials and Methods

### Collection and Maintenance of Seagrasses

*Thalassia hemprichii*, *C. serrulata* and *H. stipulacea* were collected in the dry season 2016 from different areas of a seagrass meadow located in the western coast of Zanzibar, Tanzania (6°7′43″S, 39°10′47″E). The selected meadows are within a shallow area (0.5–4 m depth) situated in the East coast of Changuu Island, located approximately 5 km northwest of Stone Town. This island is an uninhabited coral rock outcrop, although is a touristic spot due to its turtle zoo and snorkeling trips to the fringing reef surrounding the seagrass meadows. Low nutrient concentrations were observed in both the water column (NO_3_^–^ + NO_2_^–^: 0.13 ± 0.01 μM, NH_4_^+^: 0.67 ± 0.05 μM, PO_4_^3–^: 0.42 ± 0.07 μM), and in pore water at 5 cm below the sediment surface (NO_3_^–^ + NO_2_^–^: 0.42 ± 0.07 μM, NH_4_^+^: 1.36 ± 0.37 μM, PO_4_^3–^: 0.74 ± 0.11 μM). Therefore, this area is relatively pristine with the highest relative cover of seagrasses and the highest water quality within the sites included in our study in Zanzibar Archipelago (Teichberg et al., *in preparation*). Temperature in the seagrass meadows varied between 26.4 and 28.4°C with mean values of 27.30 ± 0.08°C during daytime (Teichberg et al., *in preparation*). Rhizomes with approximately 5 to 6 shoots were collected for each species from different areas around the meadow to avoid collecting shoots from the same individual plant. Seagrasses were packed in paper tissues dampened with seawater, placed inside plastic bags and transported within 48 h to the Marine Experimental facilities (MAREE) at the Leibniz Centre for Tropical Marine Research (ZMT) in Bremen (Germany). Once at the MAREE, seagrasses were replanted in polypropylene trays filled with marine carbonate substrate of at least 10 cm depth. Acclimation took place in 300-l aquaria with a recirculation system mimicking as best as possible the original natural conditions of the seagrass plants. The collected seagrasses co-inhabited the aquaria with fish, hermit crabs, sponges and natural rocks providing nutrient recycling to low nutrient (NO_3_^–^: 2.8 ± 1.5 μM, NH_4_^+^: < 0.3 μM, PO_4_^3–^: < 0.002 μM) artificial seawater (ASW), and, therefore, avoiding any extra nutrient addition. The fluorescent lights (200 ± 30 μmol photons m^–2^ s^–1^) were regulated to a photoperiod of 12:12 h, and temperature (26 ± 1°C) and salinity (35) acclimation conditions were similar to the average values observed in native meadows, providing the seagrasses time to recuperate and acclimate to the aquaria, which based on our experience, is approximately 3 months.

### Experimental Design and Setup

We conducted an experiment with a nested split-plot design (Schielzeth and Nakagawa, 2013) to study the effects of nutrient over-enrichment, elevated temperature and their interaction on the performance of adult individuals of the three tropical seagrass species *T. hemprichii, C. serrulata* and *H. stipulacea*. The experiment was carried out in the same experimental setup as Artika et al. (2020). We applied low and high levels of temperature, 26°C (LT) and 31°C (HT), respectively, and low and high levels of nutrients by adding 2 μM NH_4_NO_3_ + 0.1 μM KH_2_PO_4_ (LN) and 20 μM NH_4_NO_3_ + 2 μM KH_2_PO_4_ (HN), respectively. This resulted in 4 experimental treatments: low temperature and low nutrient concentrations (LT + LN) considered as the control, low temperature and high nutrient concentrations (LT + HN), high temperature and low nutrient concentrations (HT + LN), and high nutrient concentration and high temperature (HT + HN). Experimental temperature treatments were selected based on lower and higher average temperatures in the area. Nutrient concentrations were selected on nutrient concentrations in impacted seagrass meadows in the area, as well as in the sewage effluent in Stone Town (Zanzibar) (Teichberg et al., *in preparation*) and the nutrient concentrations found in Changuu Island, the relatively pristine site were seagrasses were collected.

The experiment was conducted under laboratory conditions in an indoor system in the MAREE (ZMT, Bremen) with a total of 24 glass aquaria (29 × 13 × 30 cm dimensions) of 10 l volume. Each aquarium was considered as a replicate, and each of the 4 treatments had 6 replicate aquaria (see experimental design in Artika et al., 2020).

The experimental temperatures were obtained by placing aquaria in larger (250 l) experimental tanks (ETs) that acted as water baths maintaining a constant experimental water temperature. Six aquaria were placed in 4 different ETs with nutrient treatments nested within the 4 ETs set at the two temperatures and with no interactions among aquaria. Water bath temperature was controlled in each ET by heaters (EHEIM, Germany) connected to an individual electronic system that was continuously regulating the temperature (± 0.2°C) by digital controllers and individual temperature probes. Air pumps were placed in each ET to ensure water movement of the water bath. The light (200 ± 20 μmol photons m^–2^ s^–1^) was provided by 2 LED lamps (Hydra Fifty-two HD, AquaIllumination^®^, Iowa) at the top of each ET and placed at the same height. A photoperiod of 12:12 h light:dark was set with sunrise and sunset simulation. Transparent PVC lids were placed on each ET to lower water evaporation.

The experimental setup was a flow through system that took water from two water reservoirs (∼115 l each) with either high or low nutrient ASW solutions. To achieve the experimental nutrient concentrations, previously dissolved stock solutions of NH_4_NO_3_ and KH_2_PO_4_ (Merck, Germany) were added to each water reservoir. Once in the water reservoir, the solution was gently mixed with fresh ASW and an air pump was placed in each water reservoir to ensure further aeration and mixing. Water reservoirs were manually emptied from any remaining water and refilled with fresh ASW every other day. Equal water flow was assured to all aquaria by using a 24-channel peristaltic pump (ISMATEC, Germany) that maintained a constant flow rate (4 ml min^–1^) to each aquarium. Water constantly overflowed from the aquaria to the water bath of the ETs ensuring constant water renewal inside the aquaria and, at the same time, ETs drained the surplus water. In each aquarium, an air pump ensured water aeration and mixing by moving water from the bottom to the top.

After the 3-month acclimation period, seagrasses with several ramets (i.e., iterating modular plant units) and no apparent damage or epiphyte cover were selected. Three selected ramets (here forward referred to as plant) of each species were cut and carefully planted in silicate sediment (∼7 cm depth) together with 3 seedlings of *Enhalus acoroides* (Artika et al., 2020) in the experimental setup previously described, where they were acclimated one more month before the start of the treatments. *T. hemprichii* and *C. serrulata* plants consisted of one shoot of 2–4 leaves, roots and a small portion of rhizome (3.6 ± 0.2 cm long). Plants of the same rhizome were randomly distributed along the treatments. *H. stipulacea* was divided in fragments of rhizome with 3–5 shoots and 8.8 ± 0.6 cm long.

After the acclimation period, temperature was elevated by increasing 1°C day^–1^ until reaching the 31°C in two random ETs. The other two ETs remained at the acclimation temperature of 26°C. Once the target temperatures were stable, nutrient addition started, and from that moment the experiment started. Plants were exposed to the selected experimental treatments for approximately 1 month (January 20th to February 22nd 2017) to ensure a response of the selected individual plant traits (Lee et al., 2007; McMahon et al., 2013; Roca et al., 2016).

### Water Monitoring and Sampling

Water pH, temperature and salinity were monitored every other day during the acclimation and experimental phase with a multi-parameter probe (WTW Multiprobe). Salinity was adjusted by adding distilled water when necessary to aquaria and water reservoirs. Hobo loggers continuously monitored water temperature inside one of the aquaria in each ET (*n* = 4). The electronic system within the ETs continuously measured the temperature of all the water baths. Water was sampled every week from the two water reservoirs and random aquaria of each treatment (*n* = 4 each week) for silicate, phosphate, and dissolved inorganic N (DIN), as the sum of NH_4_^+^, NO_3_^–^ and NO_2_^–^. Water was sampled with a syringe, immediately filtered (0.45 μm pore size, Whatman GF/F filters) in pre-rinsed polyethylene bottles and frozen (−20°C). Analysis was performed using a continuous flow injection analyzing system (Skalar SAN^++^-System) following Grasshoff et al. (1999). The measuring procedure had a relative standard deviation < 3.5% with reference to the linear regression of an equidistant 10-point calibration line from NIST standards.

Detached leaves of the seagrasses were removed from the aquaria every day, and epiphytes growing on the blades of the seagrasses were removed weekly. Microalgae growing in the chamber walls, however, were not removed.

At the end of the experiment, the sediment N and C content were analyzed by drying the homogenized samples in a forced air oven at 60°C until constant dry weight (DW), ground to a fine powder with mortar and pestle, and weighed into tin capsules prior to analysis using Euro EA3000 Elemental Analyzer. Water samples from all aquaria were sampled for chlorophyll *a* (Chl-*a*) and *b* (Chl-*b*) and suspended particulate matter (SPM) measurements. Water was immediately filtered under constant pressure onto pre-combusted (5 h, 450°C) and pre-weighed Whatman GF/F filters (0.45 μm pore size). Filters for SPM analysis were dried at 50°C and filters for Chl-*a* and *b* analysis were stored at −20°C. Concentrations of SPM were determined by weighing the dried filter, subtracting the weight of the empty filter and dividing it by the respective volume of water filtered. Pigments were extracted from the filters in 8 ml of 96% ethanol in glass vials heated for 5 min at 80°C, covered with aluminum foil, and placed in a rotor at room temperature for approximately 24 h. Extracts were subsequently centrifuged at 5000 rpm for 20 min. Chl-*a* and *b* samples were determined in a photometer Shimadzu UV-1700.

### Measurement of Seagrass Traits

Different biochemical, morphological and physiological individual-level traits were selected based on their quick response times observed under other single effect experiments of nutrient over-enrichment or temperature in order to better understand the combined effect of these factors (Campbell et al., 2006; Lee et al., 2007; Leoni et al., 2008; Martínez-Crego et al., 2008; Pedersen et al., 2016; Roca et al., 2016).

At the end of the experiments, seagrasses were removed from the aquaria and the morphological measurements on each plant were performed. Afterward, plants were carefully separated with a glass spatula into the different parts: leaves (for fluorescence measurements, nutrient content, and free amino acids content, FAAs), rhizome (for nutrient content, and non-structural carbohydrate content, NSC), and roots (for nutrient content) in *T. hemprichii* and *C. serrulata* plants. Plant tissues of the three plants of each species within each aquarium were pooled together for nutrient analysis. In *H. stipulacea* plants, due to the limited plant material, rhizome and root material were pooled, and FAAs in leaves were not measured. Moreover, samples from two aquaria of this latter species had to be pooled for fluorescence measurements and nutrient content analysis.

#### Biochemical Traits

##### Nitrogen and carbon content

To assess whether the experimental water column N enrichment affected internal nutrient storage and allocation, we measured the final N and C contents of different plant tissues. *T. hemprichii* and *C. serrulata* plants were divided into leaves, rhizome and roots; while *H. stipulacea* individuals were divided into leaves (representing the AG compartment) and rhizome and roots (representing the BG compartment). Samples were dried at 50°C in a forced air oven until constant DW, ground to a fine powder with mortar and pestle, and weighed (1.42 ± 0.02 mg) into tin capsules using an analytical scale prior to analysis using Euro EA3000 Elemental Analyzer.

##### Free amino acid content in leaves

The FAAs were extracted from ∼50 mg fresh weight (FW) of leaves of *T. hemprichii* and *C. serrulata* grounded material (FastPrep^®^-24 Instrument) during 60 min at room temperature by adding 4 ml of 0.05 N HCl. The supernatant (5 min, 10,000 *g*) was filtered through 0.2 μm CA-filters into glass vials and stored at −20°C for posterior FAAs composition and concentration analysis using an ion-exchange liquid chromatography for hydrolyzed samples (Biochrom 30). For simplicity, only the total FAA concentration (sum of 14 FAAs for these samples: ALA, ARG, ASP, GLU, GLY, HIS, ILE, LEU, MET, PHE, THR, TYR, SER and VAL) were considered in this study. However, relative differences among FAAs can also be identified (Supplementary Table 1).

##### Non-structural carbohydrate content in rhizomes

The concentration of sucrose and starch were measured on rhizome material of *T. hemprichii* and *C. serrulata* and in the BG compartment (rhizome and roots) of *H. stipulacea*. We followed a modified protocol from Salo and Pedersen (2014). The samples were frozen (−80°C) and freeze-dried for 48 h. Soluble sugars, namely sucrose, were extracted from ground plant tissue by boiling in 96% ethanol. The ethanol extracts were evaporated and the residues were dissolved in deionized water for sucrose analysis. Starch was extracted from the ethanol-insoluble residue in 1 N NaOH for 24 h. The sucrose and starch concentrations of the extracts were determined spectrophotometrically (wavelengths 486 and 640 nm, respectively) using resorcinol and anthrone assays, respectively, with sucrose as a standard (Yemm and Willis, 1954; Huber and Israel, 1982). Results were reported in sucrose equivalents g^–1^ DW. Current testing of this method has shown that NaOH extracts not only starch, but also cellulose, which can confound the results. Regarding the sucrose determination, this method only determines ketoses (as fructose) so we are ignoring glucose, the other component of sucrose, therefore underestimating the final concentrations (M. Birkicht, personal communication). Despite its drawbacks, this method has been frequently used in other studies and allows for direct comparisons of the data.

#### Morphological Traits

Morphological measurements were individually performed in each of the three plants of each species within the different aquaria. The measurements included leaf morphometrics (length, width and surface area, SA), sheath length (or petiole for *H. stipulacea*), root length, and internode length (IL) (just for *H. stipulacea*).

#### Physiological Traits

##### Growth rates

Leaves of *T. hemprichii* and *C. serrulata* were double-pinned in parallel just above the sheath at the beginning of the experiment for leaf growth measurements following the method by Short and Duarte (2001). However, leaf growth and turnover were higher than expected during the experiment, and no leaves with pinning remained at the end of the experiment. Therefore, the growth was estimated by collecting the detached leaves found daily in each aquaria. Leaf growth (cm d^–1^) was measured as the distance from the base of the detached leaf to the place where the pins were, divided by the days when the leaf was sampled. Only leaves detached during the last week of the experiment were considered. We tried to measure *H. stipulacea* growth by marking the rhizome, however, no label was found by the end of the experiment, and no optional method of growth estimation was possible with this species.

##### Photosynthetic variables

The photosynthetic performance of the seagrasses was measured through pulse amplitude modulated (PAM) chlorophyll fluorescence using rapid light response curves (RLCs) generated by the PAM-2500 chlorophyll fluorometer (Walz, Germany). RLCs were performed above the meristem of the second leaf of the three plants of each aquarium for *T. hemprichii* and *C. serrulata*. For *H. stipulacea*, 3 measurements were performed at the base of the leaf, close to the petiole, and plants of two aquaria of each treatment had to be pooled. The basal portion of the leaf was chosen since it represents similar distances from the surface (and thus from the light source) among plants with different leaf lengths, thus minimizing variability within plants and species (Winters et al., 2011). A clip was attached to the leaf and helped to hold the optical cable of the PAM at 3 mm distance from the tissue and to dark adapt the tissue during 5 min. Leaves were maintained in a petri dish with some ASW during the dark adaptation and the measurements.

The first quantum yield measurement was performed in the absence of actinic light (dark-adapted effective quantum yield, *Y*_o_; Saroussi and Beer, 2007), after which the RLC consisted of 12 saturating light pulses (separated by 30s intervals), increasing the photosynthetic active radiation (PAR) between pulses until 2000 μmol photons m^–2^ s^–1^. Each step lasted 10 s and was followed by a measurement of the effective quantum yield (Δ*F*/*F*_m_’) (Ralph and Gademann, 2005). From the data of the RLC, light saturation coefficient (E_k_) and the slope of the light limited part of the curve (Alpha) were calculated using the package Phytotools (Silsbe and Malkin, 2015) following the model of Jassby and Platt (1976) under the R software (R Core Team, 2019).

The maximum light utilization efficiency or maximum quantum yield of PSII was calculated following equation by Genty et al. (1989) [F_*v*_/F_*m*_ = (*F*_*m*_-*F*_*o*_)/*F*_*m*_], where *F*_*m*_ is the maximum dark-adapted fluorescence and *F*_*o*_ is the minimal fluorescence from a dark-adapted sample.

The relative electron transport rate (rETR) was calculated for each step of the curve following equation by Sakshaug et al. (1997), [rETR = (*F*_*m*__’_-*F’*/*F*_*m’*_)^∗^(PAR/2)], where F_m’_ is the light adapted maximum fluorescence and F’ the fluorescence yield at a particular light level. From the rETR values, maximum rETR (rETR_*m*__ax_) was estimated as the inflection point of the fitted rETR curve.

### Statistical Analysis

The experiment followed a split-plot design with three nesting factors (Schielzeth and Nakagawa, 2013). The two main factors (temperature and nutrient) had two fully crossed levels each (LT and HT, and LN and HN respectively). Two ETs were nested within each temperature treatments, six aquaria were nested within each ET and three plants of each species within each aquaria.

We used permutational multivariate analysis of variance (PERMANOVA) (Anderson et al., 2008) to analyze the data of each species. The Pseudo F-statistic was used to test the null hypothesis of no differences in the position of the group centroids in the space of the chosen dissimilarity measure. The fixed effects in the model were temperature and nutrient treatments, and their interactions, together with the nesting structure of temperature, ET and Aquarium. The factor Aquarium was not included in biochemical trait analysis because tissues from the different plants within aquaria were pooled. For *H. stipulacea* data, the factors Aquarium or ET were not included in biochemical measurements analysis because tissues from different aquaria were pooled. Data were scouted for outliers, which were identified as data exceeding 1.5 times the interquartile range of variation of the dataset. Outliers were only eliminated from the model when they did not allow to meet the model assumptions. We calculated the Euclidean dissimilarity matrix for all variables, as they were continuous. The assumptions of exchangeability of permutable units and homogeneity of multivariate dispersion were tested before analysis. When the homogeneity of multivariate dispersions was not met, the data were transformed (square root, or log) and the dissimilarity matrix recalculated. The homogeneity of multivariate dispersion assumption for ET grouping could not be met for leaf C:N ratio and Alpha values in *T. hemprichii*.

Water parameters in aquaria (DIN, phosphate, SPM, and Chl-*a* and *b*) were compared using PERMANOVA while DIN and phosphate concentrations in water reservoirs were analyzed using a two-way ANOVA. Temperature, nutrient, their interactions and the nesting structure (Temperature:ET:Aquarium) were the fixed effects in the model. The permutational unit for the model was the aquarium with 999 permutations, which is the recommended minimum number to test at an alpha-level of 0.05 (Manly, 1997). Comparisons were considered significant when *P* was ≤ 0.05. We used R software to perform the analysis (R Core Team, 2019) with the adonis2 function of the package “vegan” (Oksanen et al., 2019).

Pearson correlation analysis was applied in order to investigate potential relationships between biochemical, morphological and physiological traits. Data from aquaria of all treatments were combined to generate statistically independent means for each aquarium (without error), resulting in statistically independent replicate measurements (*n* = 24, except of *H. stipulacea*, *n* = 12). This statistical analysis was performed with SPSS (IBM SPSS Statistics for Windows v.24, Armonk, NW, United States).

## Results

### Experimental Conditions

The four different treatments, combining two nutrient and temperature levels, changed the conditions under which the seagrasses were grown. Water temperature in the aquaria was shown to be constant both during the day, with no sharp variations when light was absent (data not shown), and through the experimental period, while showing significant differences between temperature treatments (PERMANOVA, *P* < 0.01) (Table 1). DIN and phosphate concentrations in the two main water reservoirs were within the target concentrations throughout the experiment and were significantly different between the HN and LN treatments (two-way ANOVA, *P* < 0.001). However, once in the aquaria, nutrients were rapidly taken up, resulting in low inorganic nutrient concentrations in all treatments regardless of the treatment (PERMANOVA, *P* > 0.05). In fact, some of the concentrations measured were not included in the analysis as they were below the quantification limit. However, even though nutrient concentration parameters in aquaria were low, other observable parameters suggested eutrophic conditions were occurring in the HN treatments. Algal blooms were observed in the HN treatments, showing different trophic conditions in the LN and HN treatments, especially in the HT + HN treatment. Algae were observable by naked eye on the glass of the aquaria and seagrasses. However the abundance of these microorganisms could not be quantified, as they formed fluffy layers that disintegrated when tried to sample them. SPM concentrations in the water column were also higher in the HN treatments (PERMANOVA, *P* < 0.05) with the highest concentrations in the HT + HN treatment (Table 1). Water column Chl-*a* concentrations were also higher in the HN treatments (2.6–5.9 μg l^–1^) than in the LN treatments (2−2.1 μg l^–1^) showing the highest mean value in the HT + HN treatment (5.91 μg l^–1^). Even though Chl-*b* concentrations also increased in the HT + HN treatment, no significant differences were observed among treatments (PERMANOVA, *P* > 0.05). Therefore, eutrophic conditions were especially noticeable in the HT + HN treatment. The water column nutrient enrichment did not significantly change the sediment nutrient conditions, and sediment N concentrations were below the detection limits in all aquaria (data not shown). The other variables measured in the water column, including salinity, pH and Si concentrations, were constant throughout the experiment and did not show significant differences among treatments (Table 1).

**TABLE 1:** Experimental seawater parameters (mean ± SE) and number of measurements taken (n) during the experimental period in each of the four treatments (LT, low temperature; LN, low nutrient; HT, high temperature; HN, high nutrient).

		Treatments			
	*n*	LT + LN	HT + LN	LT + HN	HT + HN
DIN (μM)^ R^	4	5.66 ± 0.51		22.40 ± 0.98	
PO_4_^–^ (μM)^ R^	4	0.19 ± 0.01		1.01 ± 0.04	
Water temperature (°C)	25*	26.25 ± 0.05	31.01 ± 0.07	26.28 ± 0.03	31.01 ± 0.05
	3735^‡^	26.47 ± 0.003	31.13 ± 0.004	26.22 ± 0.004	31.48 ± 0.002
Salinity	25	35.43 ± 0.12	35.39 ± 0.10	35.33 ± 0.07	35.39 ± 0.11
pH	12	8.47 ± 0.02	8.39 ± 0.02	8.67 ± 0.03	8.59 ± 0.02
DIN (μM)	5	0.62 ± 0.33	0.88 ± 0.44	0.64 ± 0.31	1.25 ± 0.01
PO_4_^–^ (μM)	5	0.15 ± 0.01	0.14 ± 0.02	0.14 ± 0.00	0.14 ± 0.01
Si (μM)	5	0.71 ± 0.07	1.15 ± 0.22	0.91 ± 0.20	0.85 ± 0.06
SPM (mg l^–1^)	24	27.62 ± 4.65	24.04 ± 1.99	38.21 ± 3.69	74.91 ± 11.75
Chl-*a* (μg l^–1^)	24	2.07 ± 0.53	2.07 ± 0.53	2.59 ± 0.98	5.91 ± 2.41
Chl-*b* (μg l^–1^)	24	0.36 ± 0.07	0.36 ± 0.07	0.42 ± 0.18	0.88 ± 0.43

DIN, dissolved inorganic nitrogen (the sum of NH_4_^+^, NO_3_^–^ and NO_2_^–^); SPM, suspended particulate matter. ^R^Measurements taken in the water reservoirs, *Water temperature provided by multiprobe measurements, ^‡^ Water temperature provided by continuous measurement of Hobo loggers.

### Seagrass Biochemical Traits

**FIGURE 1:**
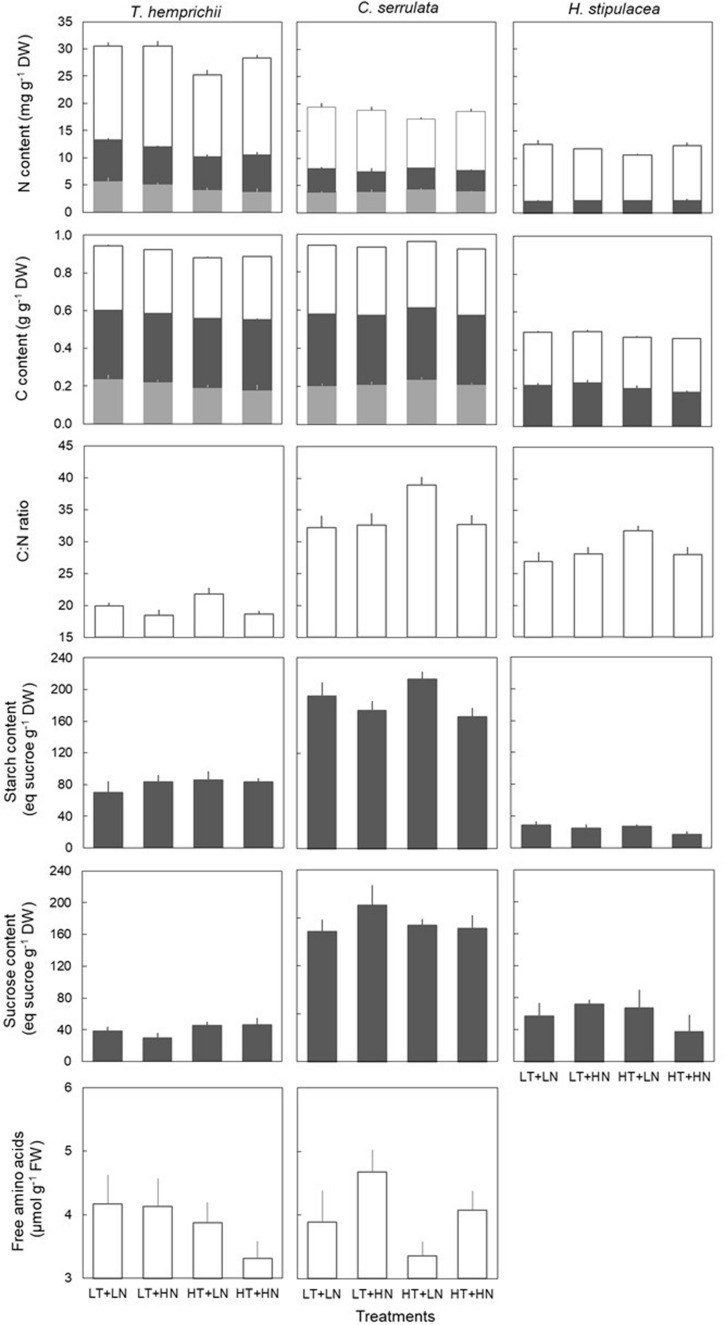
Biochemical traits (mean ± SE, *n* = 6, except of *H. stipulacea*, *n* = 3) of *T. hemprichii*, *C. serrulata* and *H. stipulacea* in leaves (open bars), rhizome (dark gray bars) and roots (light gray bars) in the four treatments (LT, low temperature; LN, low nutrient; HT, high temperature; HN, high nutrient).

*Thalassia hemprichii* showed the highest N content in all three tissues compared to that of the other species, followed by *C. serrulata* and *H. stipulacea* (Figure 1). Total C content was similar in all species, although allocation changed. While *T. hemprichii* showed higher C content in rhizome compared to leaves, *C. serrulata* showed similar content in these two tissues. On the other hand, *H. stipulacea* had slightly higher C content in AG than BG tissues (Figure 1).

Temperature treatments changed the biochemical contents and the nutrient allocation in the three species (Table 2). *C. serrulata* was the species most influenced by temperature in terms of its leaf biochemical traits, as leaf N and C content decreased, and C:N ratio significantly increased under HT treatments (Figure 1). Leaf C:N ratio also increased in *H. stipulacea*, although no significant effect was detected. *T. hemprichii* BG tissues were the most influenced by HT treatment, with significantly lower N content in rhizome and roots under HT treatments (Table 2). Root C content was also significantly lower under HT treatments in this species and *H. stipulacea*, while in *C. serrulata*, significantly higher C content was observed under HT treatments (Figure 1).

**TABLE 2:** Permutational analysis of variance (PERMANOVA) of the effects of temperature (T) and nutrient (N) treatments on the biochemical traits of *T. hemprichii*, *C. serrulata*, and *H. stipulacea* in Figure 1.

		*T. hemprichii*					*C. serrulata*					*H. stipulacea*				
Trait	Source	df	SS	*R*^2^	Pseudo-F	*P*	df	SS	*R*^2^	Pseudo-F	*P*	df	SS	*R*^2^	Pseudo-F	*P*
Leaf N	T	1	2.37	0.1	3.08	0.112	1	2.45	0.11	3.58	0.075	1	1.36	0.12	2.15	0.197
	N	1	4.86	0.21	6.32	0.031*	1	2.78	0.12	4.07	0.058*	1	0.39	0.04	0.61	0.437
	T:N	1	0.84	0.04	1.09	0.298	1	1.18	0.05	1.72	0.203	1	4.22	0.38	6.69	0.045*
	T:ET	2	1.07	0.05	0.7	0.527	2	4.29	0.19	3.14	0.069	-	-	-	-	-
	Res	18	13.85	0.6			18	12.3	0.53			8	5.04	0.46		
Rhizome N ^‡^	T	1	4.42	0.19	4.75	0.042*	1	0.07	0	0.07	0.781	1	0.09	0.01	0.07	0.789
	N	1	0.09	0.004	0.1	0.751	1	2.27	0.1	2.19	0.173	1	0.08	0.01	0.06	0.806
	T:N	1	1.44	0.06	1.55	0.223	1	1.53	0.07	1.48	0.259	1	0.04	0	0.03	0.852
	T:ET	2	0.29	0.01	0.15	0.847	2	0.51	0.02	0.25	0.814	-	-	-	-	-
	Res	18	16.76	0.73			17	17.61	0.8			8	10.78	0.98		
Roots N	T	1	8.12	0.35	16.86	0.001***	1	2.25	0.1	2.61	0.130	-	-	-	-	-
	N	1	0.83	0.04	1.72	0.223	1	0.15	0.01	0.17	0.704	-	-	-	-	-
	T:N	1	0.03	0	0.07	0.793	1	1.92	0.09	2.22	0.142	-	-	-	-	-
	T:ET	2	5.35	0.23	5.55	0.017*	2	3.02	0.14	1.75	0.207	-	-	-	-	-
	Res	18	8.67	0.38			17	14.66	0.67			-	-	-		
Leaf C:N	T	1	1.41	0.06	2.05	0.184	1	3.07	0.13	4.78	0.042*	1	2.58	0.23	4.18	0.084
	N	1	6.48	0.28	9.4	0.010**	1	2.84	0.12	4.43	0.063	1	0.78	0.07	1.26	0.303
	T:N	1	0.87	0.04	1.27	0.276	1	2.81	0.12	4.39	0.038*	1	2.72	0.25	4.42	0.054*
	T:ET	2	1.83	0.08	1.33	0.309	2	2.74	0.12	2.13	0.158	-	-	-	-	-
	Res	18	12.4	0.54			18	11.54	0.5			8	4.93	0.45		
Leaf C	T	1	4.44	0.19	5.21	0.029*	1	8.77	0.4	14.67	0.002**	1	0.04	0	0.04	0.838
	N	1	0.34	0.01	0.4	0.521	1	0.02	0	0.04	0.855	1	0.03	0	0.02	0.864
	T:N	1	2.37	0.1	2.78	0.129	1	0.47	0.02	0.78	0.358	1	2.76	0.25	2.7	0.126
	T:ET	2	0.5	0.02	0.3	0.728	2	2.58	0.12	2.16	0.136	-	-	-	-	-
	Res	18	15.35	0.67			17	10.16	0.46			8	8.17	0.74		
Rhizome C^‡^	T	1	0.11	0.005	0.1	0.792	1	0.14	0.01	0.58	0.474	1	3.96	0.36	5.3	0.045*
	N	1	0.03	0.001	0.02	0.902	1	15.01	0.68	60.44	0.001***	1	0.03	0	0.04	0.859
	T:N	1	0.22	0.01	0.2	0.690	1	0.36	0.02	1.44	0.258	1	1.04	0.09	1.39	0.265
	T:ET	2	2.7	0.12	1.22	0.339	2	2.26	0.1	4.55	0.028*	-	-	-	-	-
	Res	18	19.95	0.87			17	4.22	0.19			8	5.97	0.54		
Roots C	T	1	5.04	0.22	8.97	0.010**	1	3.12	0.14	4.7	0.034*	-	-	-	-	-
	N	1	0.67	0.03	1.2	0.287	1	0.65	0.03	0.99	0.383	-	-	-	-	-
	T:N	1	0.01	0.0003	0.01	0.907	1	3.01	0.14	4.53	0.041*	-	-	-	-	-
	T:ET	2	7.18	0.31	6.4	0.011*	2	3.93	0.18	2.96	0.067	-	-	-	-	-
	Res	18	10.1	0.44			17	11.29	0.51			-	-	-		
Rhizome sucrose^‡^	T	1	2.21	0.1	2.01	0.165	1	0.32	0.01	0.28	0.606	1	0.34	0.03	0.31	0.578
	N	1	0.25	0.01	0.23	0.684	1	0.79	0.04	0.71	0.392	1	0.09	0.01	0.08	0.774
	T:N	1	0.34	0.02	0.31	0.606	1	1.46	0.07	1.3	0.230	1	1.78	0.18	1.6	0.263
	T:ET	2	0.54	0.02	0.25	0.794	2	0.46	0.02	0.21	0.801	-	-	-	-	-
	Res	17	18.65	0.85			17	18.98	0.86			7	7.79	0.78		
Rhizome starch^‡^	T	1	0.69	0.03	0.81	0.389	1	0.26	0.01	0.34	0.563	1	0.54	0.05	0.58	0.475
	N	1	0.31	0.01	0.36	0.567	1	5.23	0.23	6.81	0.020*	1	2.39	0.24	2.58	0.145
	T:N	1	0.67	0.03	0.79	0.388	1	1.05	0.05	1.36	0.259	1	0.57	0.06	0.62	0.454
	T:ET	2	6.1	0.27	3.61	0.052*	2	2.64	0.11	1.72	0.22	-	-	-	-	-
	Res	18	15.23	0.66			18	13.82	0.6			7	6.49	0.65		
Leaf FAA	T	1	2.13	0.09	2.24	0.169	1	2.15	0.09	2.3	0.162	-	-	-	-	-
	N	1	0.61	0.03	0.64	0.454	1	3.8	0.17	4.07	0.068	-	-	-	-	-
	T:N	1	0.48	0.02	0.5	0.475	1	0.01	0	0.01	0.917	-	-	-	-	-
	T:ET	2	2.65	0.12	1.39	0.283	2	0.22	0.01	0.12	0.891	-	-	-	-	-
	Res	18	17.13	0.74			18	16.82	0.73			-	-	-		

Significant differences are in boldface (*: ≤ 0.05; **: ≤ 0.01; ***: ≤ 0.001). (ET: experimental tank, FAA: free amino acids). ^‡^Below-ground tissues (rhizome and roots) for H. stipulacea.

Nutrient treatments significantly influenced the nutrient contents of *T. hemprichii* and *C. serrulata* but not of *H. stipulacea* (Table 2). Both *T. hemprichii* and *C. serrulata* showed significantly higher leaf N content under HN treatments, and *T. hemprichii* also showed significantly lower C:N ratio (*P* < 0.05), while non-significant decreasing values were observed in *C. serrulata* (*P* = 0.06). Leaf FAAs content only positively responded to nutrient treatment in *C. serrulata* (*P* = 0.06). This species also showed the highest concentrations of NSC in the rhizome of the three species. In *C. serrulata* and *H. stipulacea*, the main NSC form was sucrose, while in *T. hemprichii*, starch concentrations were higher than sucrose under all treatments. The BG tissues of *C. serrulata* were the only ones that changed in response to nutrient enrichment, with lower starch and C content in rhizomes, and lower C content in roots in HN treatments (Figure 1 and Table 2).

The effects of warming on *C. serrulata* and *H. stipulacea* biochemical traits, namely leaf C:N ratio in both species and root C content in *C. serrulata*, were significantly mediated by nutrient over-enrichment. This resulted in an antagonistic effect of both factors, as values were lower than expected for effects to be additive. Contrary, N content in AG tissues in *H. stipulacea* showed the only synergistic effect observed in this study. There were no interactive effects in the biochemical traits of *T. hemprichii* (Table 2).

### Seagrass Morphological Traits

**FIGURE 2:**
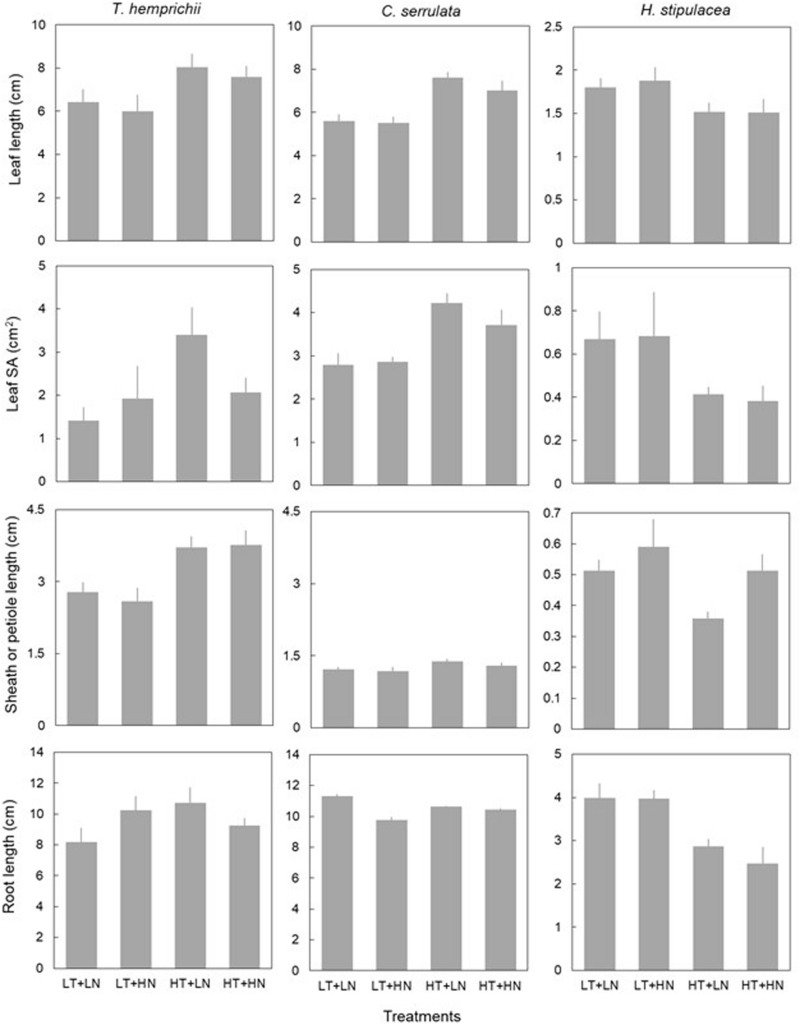
Morphological traits (mean ± SE, *n* = 6) of *T. hemprichii*, *C. serrulata* and *H. stipulacea* in the four treatments (LT, low temperature; LN, low nutrient; HT, high temperature; HN, high nutrient). Note that *Y*-axis of *H. stipulacea* panels differ.

Temperature had an overall significant effect on seagrass morphological traits (Figure 2 and Table 3). Leaf morphology was enhanced under HT treatments in *T. hemprichii* and *C. serrulata*, with longer leaves, bigger leaf SA, and longer sheaths. Root length did not respond to HT treatment in any of these two species. However, HT negatively influenced *H. stipulacea* leaf traits, with shorter leaves and smaller leaf SA, and also shorter roots and IL (Figure 2 and Supplementary Figure 1).

**TABLE 3:** Permutational analysis of variance (PERMANOVA) of the effects of temperature (T) and nutrient (N) treatments on the morphological traits of *T. hemprichii*, *C. serrulata*, and *H. stipulacea* in Figure 2 and Supplementary Figure 1.

		*T. hemprichii*					*C. serrulata*					*H. stipulacea*				
Trait	Source	df	SS	*R*^2^	Pseudo-F	*P*	df	SS	*R*^2^	Pseudo-F	*P*	df	SS	*R*^2^	Pseudo-F	*P*
Leaf length	T	1	9.12	0.13	12.97	0.002**	1	16.19	0.23	18.39	0.001***	1	7.97	0.13	13.06	0.001***
	N	1	0.63	0.01	0.9	0.349	1	0.6	0.01	0.68	0.404	1	0.92	0.01	1.51	0.246
	T:N	1	0	0	0	0.969	1	0.33	0	0.37	0.536	1	0.07	0	0.11	0.753
	T:ET:Aq	20	27.5	0.39	1.96	0.028*	20	11.65	0.16	0.66	0.86	20	29.65	0.47	2.43	0.008**
	Res	48	33.75	0.48			48	42.24	0.59			40	24.4	0.39		
Leaf SA	T	1	11.06	0.16	33.35	0.001***	1	12.19	0.17	13.11	0.001***	1	5.78	0.09	7.22	0.008**
	N	1	1.54	0.02	4.66	0.035*	1	0.46	0.01	0.49	0.478	1	0.08	0	0.1	0.757
	T:N	1	5.24	0.07	15.79	0.001***	1	0.81	0.01	0.87	0.364	1	0.07	0	0.09	0.795
	T:ET:Aq	20	37.23	0.52	5.61	0.001***	20	12.92	0.18	0.7	0.794	20	25.06	0.4	1.57	0.138
	Res	48	15.92	0.22			48	44.63	0.63			40	32.01	0.51		
Sheath length^‡^	T	1	17.93	0.25	27.47	0.001***	1	5.43	0.08	6.54	0.016*	1	2.26	0.04	2.44	0.131
	N	1	0.08	0	0.12	0.711	1	1	0.01	1.2	0.266	1	1.4	0.02	1.51	0.253
	T:N	1	0.24	0	0.37	0.554	1	0.27	0	0.33	0.57	1	2.97	0.05	3.2	0.074
	T:ET:Aq	20	21.43	0.3	1.64	0.085	20	24.49	0.34	1.48	0.135	20	19.31	0.31	1.04	0.323
	Res	48	31.33	0.44			48	39.81	0.56			40	37.07	0.59		
Root length	T	1	1.49	0.02	2.39	0.138	1	0	0	0	0.994	1	23.51	0.37	46.39	0.001***
	N	1	0.48	0.01	0.77	0.37	1	1.21	0.02	1.3	0.254	1	0.59	0.01	1.16	0.316
	T:N	1	7.19	0.1	11.52	0.002**	1	0.73	0.01	0.78	0.387	1	0.04	0	0.09	0.776
	T:ET:Aq	20	31.88	0.45	2.55	0.006**	20	24.33	0.34	1.3	0.245	20	18.59	0.3	1.83	0.058*
	Res	48	29.96	0.42			48	44.74	0.63			40	20.27	0.32		
IL	T	-	-	-	-	-	-	-	-	-	-	1	3.44	0.05	3.85	0.054*
	N	-	-	-	-	-	-	-	-	-	-	1	3.29	0.05	3.69	0.069
	T:N	-	-	-	-	-	-	-	-	-	-	1	1.07	0.02	1.2	0.262
	T:ET:Aq	-	-	-	-	-	-	-	-	-	-	20	19.47	0.31	1.09	0.392
	Res	-	-	-			-	-	-			40	35.73	0.57		

Significant differences are in boldface (*: ≤ 0.05; **: ≤ 0.01; ***: ≤ 0.001). (ET: experimental tank, Aq: aquarium, SA: surface area, IL: internode length). ^‡^Petiole length for H. stipulacea.

Overall, nutrient addition did not show significant effects on morphological trait responses with the exception of lower leaf SA of *T. hemprichii* under HN treatments (Figure 2). *C. serrulata* morphological traits were unaffected by single nutrient treatments (Table 3).

Leaf SA and root length in *T. hemprichii* showed a significant interaction of temperature and nutrients, in which both traits showed an antagonistic effect, i.e., a cancelation of the enhanced effects of nutrients when also exposed to higher temperatures (Figure 2 and Table 3). Petiole length in *H. stipulacea* showed some additive interaction of both factors, although the effect was not significant (Table 3). *C. serrulata* showed no interactive effects of both factors in the morphological traits considered (Table 3).

Morphological variables were also significantly affected by the nested blocking variables in *T. hemprichii* and *H. stipulacea*, but not in *C. serrulata* (Table 3). Therefore, the high variability between enclosures may have confounded some effects of the temperature and nutrient treatments.

### Seagrass Physiological Traits

**FIGURE 3:**
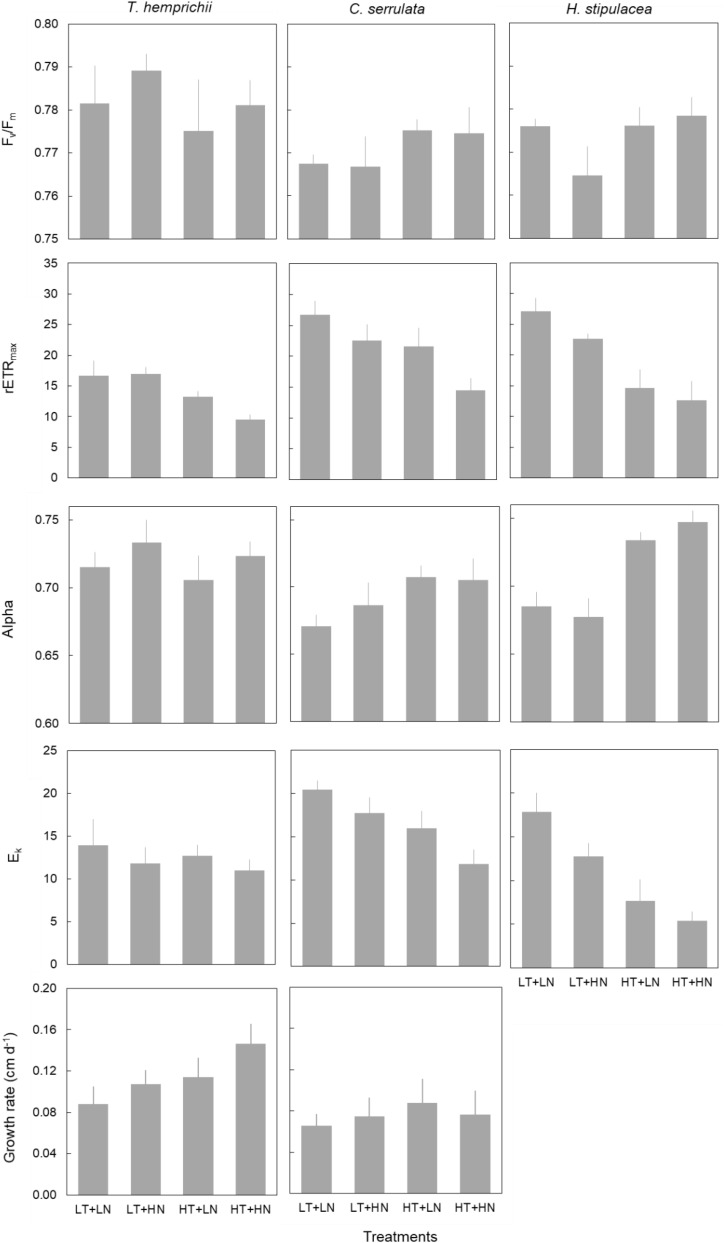
Physiological traits (mean ± SE, *n* = 6, except of *H. stipulacea*, *n* = 3) of *T. hemprichii*, *C. serrulata* and *H. stipulacea* in the four treatments (LT, low temperature; LN, low nutrient; HT, high temperature; HN, high nutrient).

Growth rate responded both to temperature and nutrient treatments in *T. hemprichii* but showed no significant effects in *C. serrulata* (Figure 3 and Table 4). On the contrary, the three species in this study significantly responded to both nutrient and temperature in terms of their photosynthetic efficiency (Figure 3 and Table 4). Temperature was the main driver of these responses, and higher F_*v*_/F_*m*_ and Alpha in *C. serrulata*, and Alpha in *H. stipulacea* were observed under HT treatments. In contrast, rETR_*max*_ was negatively affected by HT treatments in the three species, and E_*k*_ in *C. serrulata* and *H. stipulacea* (Figure 3).

**TABLE 4:** Permutational analysis of variance (PERMANOVA) of the effects of temperature (T) and nutrient (N) treatments on the physiological traits of *T. hemprichii*, *C. serrulata*, and *H. stipulacea* in Figure 3.

		*T. hemprichii*					*C. serrulata*					*H. stipulacea*				
Trait	Source	df	SS	*R*^2^	Pseudo-F	*P*	df	SS	*R*^2^	Pseudo-F	*P*	df	SS	*R*^2^	Pseudo-F	*P*
F_*v*_/F_*m*_	T	1	1	0.01	1.42	0.254	1	3	0.04	3.68	0.050*	1	1.12	0.03	1.08	0.319
	N	1	0.91	0.01	1.28	0.291	1	0.93	0.01	1.14	0.282	1	0.01	0	0.01	0.911
	T:N	1	0	0	0	0.997	1	0.05	0	0.06	0.796	1	1.48	0.04	1.43	0.234
	T:ET:Aq	20	34.59	0.5	2.45	0.020*	20	27.55	0.4	1.69	0.068	7	6.45	0.19	0.89	0.55
	Res	46	32.51	0.47			46	37.47	0.54			24	24.94	0.73		
rETR_*max*_	T	1	12.27	0.18	17.26	0.002**	1	7.61	0.11	9.78	0.002**	1	7.56	0.22	8.46	0.006**
	N	1	1.93	0.03	2.72	0.100	1	0.58	0.01	0.74	0.415	1	0.19	0.01	0.21	0.649
	T:N	1	1.25	0.02	1.75	0.193	1	1.09	0.02	1.4	0.258	1	0.01	0	0.01	0.95
	T:ET:Aq	20	20.83	0.3	1.46	0.162	20	23.93	0.35	1.54	0.108	7	4.8	0.14	0.77	0.627
	Res	46	32.72	0.47			46	35.79	0.52			24	21.45	0.63		
Alpha	T	1	0.37	0.01	0.76	0.387	1	4.56	0.07	7.81	0.004**	1	17.72	0.52	36.49	0.001***
	N	1	2.48	0.04	5.11	0.034*	1	0.02	0	0.03	0.848	1	0.29	0.01	0.6	0.476
	T:N	1	0.21	0	0.44	0.502	1	1.62	0.02	2.77	0.099	1	0.59	0.02	1.21	0.304
	T:ET:Aq	20	43.65	0.63	4.5	0.001***	20	35.91	0.52	3.07	0.001***	7	3.74	0.11	1.1	0.389
	Res	46	22.3	0.32			46	26.89	0.39			24	11.66	0.34		
E_*k*_	T	1	1.13	0.02	1.68	0.184	1	6.35	0.09	8.48	0.007**	1	19.77	0.58	46.88	0.001***
	N	1	0.97	0.014	1.45	0.275	1	2.29	0.03	3.05	0.084	1	1.01	0.03	2.41	0.135
	T:N	1	0.05	0	0.07	0.806	1	1.37	0.02	1.83	0.181	1	0.21	0.01	0.51	0.472
	T:ET:Aq	20	35.95	0.52	2.68	0.011*	20	24.55	0.36	1.64	0.091	7	2.89	0.08	0.98	0.469
	Res	46	30.9	0.45			46	34.45	0.5			24	10.12	0.3		
Growth	T	1	4.7	0.2	7.19	0.014*	1	0.35	0.02	0.31	0.563	-	-	-	-	-
	N	1	3.19	0.14	4.87	0.043*	1	0.02	0	0.02	0.902	-	-	-	-	-
	T:N	1	0.21	0.01	0.32	0.59	1	0.17	0.01	0.15	0.677	-	-	-	-	-
	T:ET	2	3.15	0.14	2.41	0.125	2	2.56	0.11	1.16	0.326	-	-	-	-	-
	Res	18	11.76	0.51			18	19.91	0.87			-	-	-		

Significant differences are in boldface (*: ≤ 0.05; **: ≤ 0.01; ***: ≤ 0.001). (ET: experimental tank, Aq: aquarium).

A single nutrient effect on photosynthetic performance was more limited than temperature alone. A significant effect of HN treatments was only detected by Alpha values in *T. hemprichii*, which was more evident in the combined HN + HT treatment (Figure 3). In *C. serrulata*, no trait showed a response to nutrients. The absence of responses of photosynthetic traits to nutrients was more evident when considering its interaction with temperature, as no significant effects were detected (Table 4). In the same way, no interaction between factors was observed in the other species in any of the physiological traits considered (Table 4).

Some photosynthetic variables, F_*v*_/F_*m*_, Alpha and E_*k*_, were significantly affected by the nested blocking variables in *T. hemprichii* and *C. serrulata* (Table 4). Therefore, the high variability between enclosures may have confounded some effects of the temperature and nutrient treatments.

### Relationships Between Biochemical, Morphological, and Physiological Seagrass Traits

**FIGURE 4:**
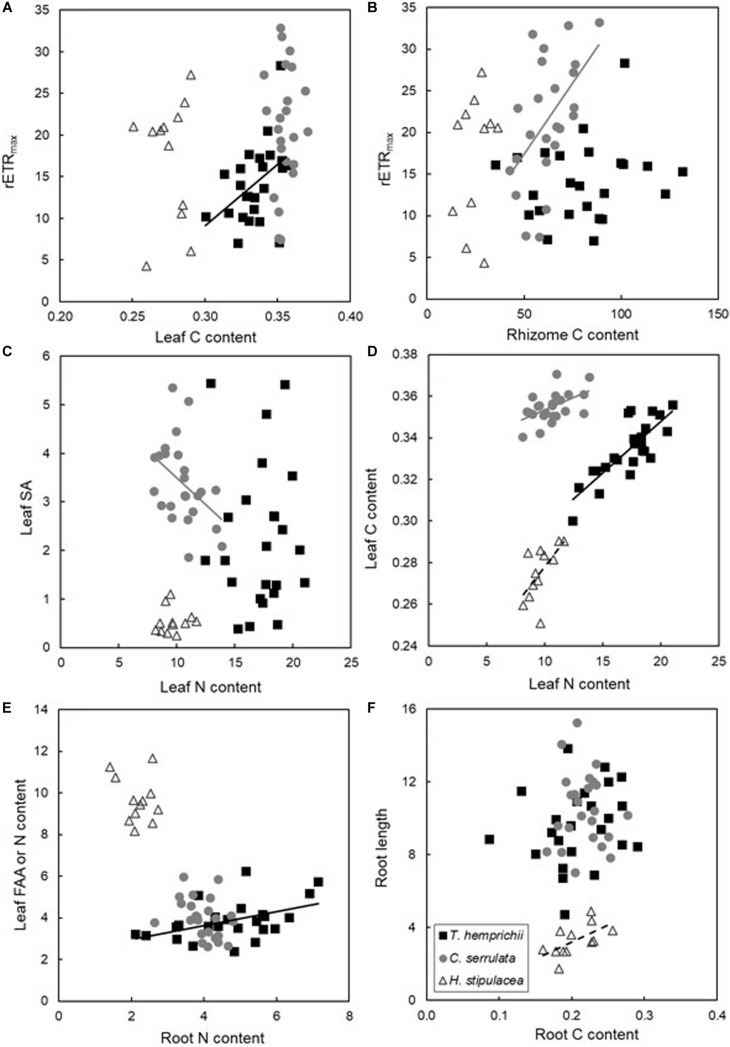
Pearson ρ correlations among selected biochemical **(A–F)**, morphological **(C,F)** and physiological **(A,B)** traits (Supplementary Tables 2–4). Correlation lines are shown only when significant as black, gray and dashed lines for *T. hemprichii, C. serrulata* and *H. stipulacea* data respectively. Panel **(E)** show leaf FAA content excepting for *H. stipulacea* that shows leaf N content.

We explored all correlations among biochemical, morphological and physiological traits within each of the three studied species (Supplementary Tables 2–4). After examining the data we recognize some correlations that were interesting to understand how trait responses differed among the three species (Figure 4).

*Thalassia hemprichii* showed no correlations between photosynthetic traits and growth, while leaf nutrient content was positively correlated with those traits (Supplementary Table 2). Specifically, leaf C content was positively correlated with _*r*_ETR_*max*_ (Figure 4A). *T. hemprichii* was also the only species showing positive correlations between AG and BG traits, both through its nutrient content and photo-physiological traits (Supplementary Table 2), such as, for instance, between FAAs in leaves and the root N (Figure 4E).

*Cymodocea serrulata* showed positive correlations among physiological traits, while negative correlations between the AG and BG nutrient contents were observed (Supplementary Table 3). Particularly, leaf nutrient contents were negatively correlated with leaf morphological traits (Figure 4C), but in terms of photosynthesis or growth, no relations with leaf physiology were detected (Figure 4A). On the contrary, photosynthetic traits were positively correlated with leaf morphology and rhizome nutrient content. For example, rETR_max_ and rhizome C content were positively correlated (Figure 4B).

Contrary to the other two species, all *H. stipulacea* correlations, when observed, were positive (Supplementary Table 4). Correlations between leaf length and BG C content, including sucrose, and E_*k*_ and root length were detected, linking AG and BG traits (Supplementary Table 4). Correlations between the same plant part were frequent, such as between leaf N and C contents (Figure 4D), or root C content and length (Figure 4F). But no correlations between photosynthetic performance and leaf morphometrics or nutrient content were detected (Figures 4A,C).

## Discussion

**FIGURE 5:**
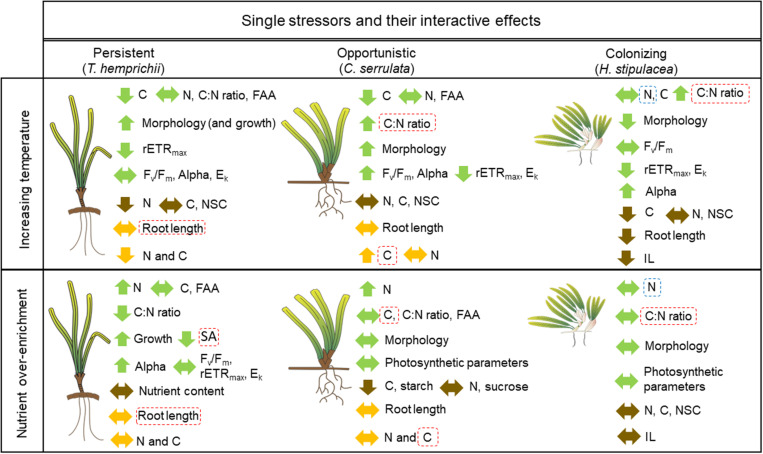
Schematic representation of the significant responses (*P* ≤ 0.05) to increasing temperature (31°C), nutrient over-enrichment and their antagonistic (dashed red lines) and synergistic (dashed blue line) interactive effects of leaves (green arrows), rhizome (brown arrows) and roots (orange arrows) in *T. hemprichii, C. serrulata* and *H. stipulacea*. Upward arrows show enhanced, downward arrows show depressed and horizontal arrows show no changes in the responses under single factor effects. (FAA, free amino acids; SA, surface area; NSC, non-structural carbohydrates; IL, internode length) Seagrass drawings are courtesy of the Integration and Application Network (www.ian.umces.edu/symbols/).

The present study aimed to examine the interactive and single effects on an extended exposure to the current ambient maximum temperature and nutrient enrichment in three tropical seagrass species. During the 5-week period, the two factors and their interaction differentially affected the biochemical, morphological and physiological traits considered, highlighting the varying strategies and tolerances among species to the treatments (Figure 5). Therefore, this study shows that *T. hemprichii*, *C. serrulata* and *H. stipulacea* have distinctly different responses to increasing temperature and nutrient enrichment despite their overlap in their distribution and the co-inhabitance of seagrass meadows. This suggests that ecosystem functioning and seagrass survivorship will be differently affected by changing environments.

### Seagrass Responses to an Extended Exposure to the Maximum Ambient Temperature

Temperature was the main driver of seagrass responses during this study, as all trait categories showed some response to the single effect of this factor, and these responses showed overall bigger differences than responses to nutrients (Figure 5). In accordance with previous studies we did not observe any variation in F_*v*_/F_*m*_ (maximum quantum yield) from reference values in any of the three species, showing that the highest target temperature (31°C) is lower than the maximum tolerance limits of the studied species (Campbell et al., 2006; Pedersen et al., 2016; Collier et al., 2017; Anton et al., 2020). However, the photosynthetic capacity (rETR_max_), related with the investment in biochemical mechanisms for CO_2_ fixation, is lower under HT treatments in the three species studied, showing some down-regulating mechanisms, and suggesting that all individuals were above their thermal optima.

In this study, *C. serrulata* and *H. stipulacea* showed photo-acclimation mechanisms to maximize C fixation and obtain extra energy, with lower saturating irradiance (E_*k*_) and maximal photosynthetic efficiency (Alpha), as shown in congeneric species (Torquemada et al., 2005; Campbell et al., 2007). Contrary to this, and in accordance with previous studies, no physiological acclimation was observed in *T. hemprichii* under HT treatments (Campbell et al., 2006; Pollard and Greenway, 2013; George et al., 2018). Instead, this latter species showed a high morphological plasticity (longer leaves and sheaths and faster growth rate) that might maximize exposure of photosynthetic material to irradiance and minimize boundary layer thickness for gas, lowering its thermal tolerance. The ability of *T. hemprichii* to change its morphology without enhancing its photosynthetic performance, suggests this species my rely on its high N storage capacity (Viana et al., 2019a) shown by the high plant N content in comparison to the other two species (Figure 1). Similar to *T. hemprichii*, the opportunistic species *C. serrulata*, also enhanced its morphology during the study period. But contrary to the former species, *C. serrulata* was able to obtain greater energy from photosynthesis suggesting that the thermal limit of this species is higher than for *T. hemprichii*. While thermal optima values in the literature are highly variable even within species, our results are in accordance with previous findings in *T. hemprichii* from the Red Sea in which the optimum temperature for the metabolic rates was 30.4°C (Anton et al., 2020). Instead, experiments with *C. serrulata* showed large declines at 35°C or lower temperatures at an individual and community level (Collier et al., 2018; George et al., 2018; Burkholz et al., 2019).

The results in this study showed that the tolerant species *H. stipulacea* also acclimated to HT treatments similar to *C. serrulata*. This acclimation was performed both by increasing its Alpha values and, contrary to *C. serrulata*, with smaller-sized plants (lower leaf and root length). The decrease in BG growth is a common strategy in seagrasses plants to adjust their productivity to environmental resources (Alcoverro et al., 2001). Shorter IL, as a proxy of higher shoot and root density, is a common feature under stressful environmental factors, suggesting that *H. stipulacea* invests excess energy (increased Alpha values) in increasing number of shoots (Jensen and Bell, 2001; Kilminster et al., 2008) rather than on leaf length. In the literature, *H. stipulacea* thermal optima and limits showed differences between populations (Nguyen et al., 2020; Wesselmann et al., 2020) but thermal optima at the Red Sea was set at 30°C (Anton et al., 2020; Wesselmann et al., 2020). It is possible that these differences highlight the importance of the different population responses in coping with stressors (McMillan and Phillips, 1979; Winters et al., 2011; Nguyen et al., 2020; Wesselmann et al., 2020) where other environmental factors, such as light irradiance, or population genetics influence the responses (Collier et al., 2016).

Therefore, projections under future climate change scenarios might vary among co-habiting species, and within the same species, depending on the local adaptations and acclimation of seagrasses across geographical ranges. Morphological plasticity was a key acclimation response of the three species, but *H. stipulacea* and *C. serrulata* were the species that better acclimated to HT temperatures by primarily changing a combination of their biochemical, physiological and morphological traits. *T. hemprichii*, a climax species, which is expected to show slower acclimation times, surprisingly acclimatized to new environmental conditions by varying primarily its morphology, in the same time frame as *H. stipulacea*, a colonizing species. Further studies on *T. hemprichii* physiology are needed to understand its acclimation strategy and high plasticity.

### Seagrass Responses to Water Column Nutrient Enrichment

In tropical environments, nutrients, as N, P or even Fe, are usually the limiting factor to growth (Pérez et al., 1991; Duarte et al., 1995; Agawin et al., 1996). Even though native species from these areas are adapted to survive in extremely low nutrient habitats, they usually respond to *in situ* or experimental nutrient additions by increasing their nutrient content, but also in terms of physiology and morphology, as shown in this study (Figure 5).

Although there were no changes in N content in the BG tissues, the enhanced leaf N content in *T. hemprichii* and *C. serrulata* suggests that they were nutrient limited. These differences between tissues might be explained because we enriched the water column but not the sediment, so it would take more time for the sediment to become nutrient enriched and to see changes in N storage in BG tissues. In contrast, the absence of biochemical and other trait responses both in AG and BG tissues of *H. stipulacea* suggests that this species was not N-limited. This interesting result might be related to the smaller size of *H. stipulacea*, which is related to the lower N demands, with lower N uptake and assimilation rates compared to the other two species, even under high N treatments (Alexandre et al., 2014; Viana et al., 2019a). But, surprisingly, *H. stipulacea* has been spotted in highly eutrophic areas where native species are not able to grow (Van Tussenbroek et al., 2016; Winters et al., 2020) showing significantly higher leaf N content and enhanced morphology compared to control sites (Mejia et al., 2016; Beca-Carretero et al., 2020). These differential trait responses between natural populations and this study suggests that trait’s plasticity under nutrient-enrichment might be an adaptative response of the population to long-term changes rather than an acclimation response at an individual level. Although interspecific competition was not purposely studied, due to the experimental design in which all seagrasses were planted together, we cannot rule out competition among the primary producers in the aquaria for nutrient resources (Artika et al., 2020). *H. stipulacea* may be outcompeted for resources due to its smaller size when compared to the leaf and sheath length of *T. hemprichii* and *C. serrulata.* However, there are several occasions where the species forms mixed meadows with other seagrass species or macroalgae (Boudouresque et al., 2009; Sghaier et al., 2011; Gambi et al., 2018; Apostolaki et al., 2019).

As N and P are normally the limiting nutrients in the tropics, C could potentially become the limiting factor under HN scenarios (Buapet et al., 2013; Apostolaki et al., 2014). Therefore, species with large C storage pools, such as *T. hemprichii*, might have their C demands already covered in all tissues (including leaves), and nutrient over-enrichment might first increase its photosynthetic efficiency (Alpha) and afterward enhance growth rate without C being limiting. Contrary to *T. hemprichii*, the opportunistic species *C. serrulata* followed a common strategy that mobilizes the rhizome carbohydrate reserves to perform photosynthesis (Figure 4B) and synthesize amino acids leading to lower levels of starch in BG tissues (Invers et al., 2004; Leoni et al., 2008). The correlation between rETR_max_ and leaf C in *T. hemprichii* (Figure 4A) also supports the hypothesis that this species does not depend on storage tissues. Together with C storage depletion, *C. serrulata* showed no enhanced growth, and lower saturating irradiance (E_*k*_), which are all typical responses to nutrient inputs in both tropical and temperate seagrasses (Martínez-Crego et al., 2008; Jiang et al., 2013).

Overall, *T. hemprichii* performed better than *C. serrulata* under short-term nutrient enrichment (> 5 weeks), while *H. stipulacea* showed a lack of response, which indicates a dormancy state typical for this tolerant species (Apostolaki et al., 2018; Hernández-Delgado et al., 2020).

### Combined Effect of Different Drivers: Stressors Do Not Act Alone

Our results show that the interaction between both factors is limited to responses in morphological and biochemical traits, as no changes were observed in the physiological performance of the seagrasses, at least during the short-term experimental period (Figure 5). This study provides the first insights of the interactive effects of nutrient over-enrichment and increasing temperature in tropical species, therefore comparisons with other tropical species of other geographic areas is still not possible. While synergistic effects are frequent in coastal ecosystems, including seagrasses, in this study we mainly detected antagonistic interactive effects of the studied stressors (Gunderson et al., 2016; Stockbridge et al., 2020). Therefore, the results within this study show that the impact of increasing temperature average values will potentially be less detrimental to plant traits in tropical nutrient over-enrichment seagrass meadows in the short-term. However, this study does not take into account other biogeochemical processes taken place during the eutrophication process, as sediment anoxia or light deprivation (Burkholder et al., 2007).

Interactions between both factors were observed in both AG and BG tissues. The effects of warming on *C. serrulata* and *H. stipulacea* leaf C:N ratio were significantly mediated by nutrient over-enrichment, showing lower values than expected if effects would be additive (Figure 1). The enhanced productivity caused by higher temperatures in these species suggests that C limitation may occur in both AG and BG tissues when other nutrients, namely N and P, are not limiting in the water column. This would not be deleterious for these species, as C:N ratio values observed under the HT + HN treatment were, overall, closer to values in the control (LT + LN treatment) showing less unbalances among tissues than it would be expected under synergistic interactions. While leaf N content in *H. stipulacea* did not respond to any single-factor treatment, it showed the only synergistic interactive effect in this study. This shows that temperature might mediate responses to N over-enrichment through enhancing N metabolism, as shown for the temperate species *Z. marina* (Alexandre et al., 2020). In the literature, biochemical responses to the interactive effect of temperature and nutrients are variable, and have also been observed in leaves and BG nutrient content of *Z. marina* (Touchette and Burkholder, 2002; Moreno-Marín et al., 2018), but not in *C. nodosa*, or other *Zostera* species (Kaldy, 2014; Brodeur et al., 2015; Egea et al., 2018; Mvungi and Pillay, 2019; Ontoria et al., 2019b).

The interactive effects of both factors in *T. hemprichii*, contrary to the results of the other two species, were related to morphological traits. Leaf SA and root length values under the HT + HN treatment decreased drastically, showing an antagonistic effect of both factors. Morphological plasticity has been suggested to have adaptive advantages in heterogeneous environments, allowing organisms to maximize resource acquisition under unpredictable or changing levels of resource availability (Houston and McNamara, 1992). In this sense, the high plasticity shown by this species, both under single and cumulative stressors, is highly remarkable, especially when compared to *C. serrulata*, indicating that *T. hemprichii* can adopt a functional role closer to opportunistic or climax species when necessary. Interactive effects on morphology have been observed in leaf SA of *Z. marina*; leaf length of *Z. capensis* (Bintz et al., 2003; Mvungi and Pillay, 2019), and more frequently under natural conditions in *T. hemprichii* and other tropical species (Udy et al., 1999; Ali et al., 2018). Even though the mechanisms behind this interaction cannot be elucidated within the current study, high physiological activity in terms of photosynthesis and nutrient uptake and assimilation under enhanced temperature and nutrient over-enrichment (Touchette and Burkholder, 2007; Kaldy, 2014; Alexandre et al., 2020) may cause this limitation of energy to enhance growth, and therefore, morphology. Also, increasing organic matter in sediment has been related with lower BG growth (Ontoria et al., 2019b), although the low nutrient concentrations in sediment in this experiment does not sustain this hypothesis as the main reason. The interaction of both factors might be positive for the species survival, as no large differences in morphological traits might infer smaller differences between AG and BG biomass, one of the main reasons behind the increasing mortalities in seagrass meadows (Collier and Waycott, 2014).

In this study, physiological traits showed little variance under combined stressors in the three species studied. These results are in accordance with other studies with temperate species that showed no interactive effects on fluorescence parameters or growth (Bintz et al., 2003; Moreno-Marín et al., 2018; Ontoria et al., 2019b). However, in some studies significant interactions were found for yield, growth (Kaldy, 2014; Mvungi and Pillay, 2019; Ontoria et al., 2019a), or photosynthetic rate, as well as gross production rates (Egea et al., 2018; Moreno-Marín et al., 2018). Comparison among studies is difficult due to the variable traits measured; and care must be taken when comparing results that differ in exposure period, the intensity of the stressors (e.g., nutrient concentration used), or even the different geographical areas of the species, as all these factors, among others, might affects the plasticity of seagrass traits.

It is generally accepted that plant acclimation follows a sequence of ordered changes that starts in the photosynthetic apparatus, followed by biochemical changes, as nutrient content, morphological changes and finally the population and community changes (McMahon et al., 2013; Roca et al., 2016). Such sequence will be also slower in foundation or persistent species, which are recognized to show higher physiological resistance than opportunistic or tolerant species. The simultaneous study of species with different life-history traits and trait categories have shown that different species might show different response and acclimation strategies. Therefore, despite the increasing number of studies on interactive effects of stressors, this study shows the need of further research to extract further conclusions on how seagrass meadows will respond to multiple stressors.

### Ecological Implications

The varying responses of the three tropical species studied might imply that there are winners and losers under the changing scenarios studied. For instance, changes in biochemical traits have been observed under single-factor and cumulative treatments, especially in the large-blade species in this study. AG nutrient changes have been related with changes in palatability and herbivory preferences which might affect the food web structure and top-down pressure, affecting the resilience capacity of seagrass meadows (Jiménez-Ramos et al., 2017). Also, although C concentrations in AG tissues could be unaffected by changes in environmental nutrient concentrations (Brodeur et al., 2015), increases in C content with increasing dissolved N or P can occur (Lee and Dunton, 1999; Ali et al., 2018; Mvungi and Pillay, 2019) (Figure 4D). In fact, the ability of *T. hemprichii* to take advantage of the excess nutrients by increasing its photosynthetic efficiency suggests a dependence between nutrient limitation and photosynthetic C incorporation related to the synthesis of photosynthetically involved molecules such as chlorophyll, on nutrient supply (Agawin et al., 1996). This can result in a biochemical unbalance caused by the allocation of photosynthetically fixed C to leaf production at the expense of nutrient-deplete BG tissues.

The diminishing C concentrations in rhizome, as observed in *C. serrulata* under HN treatments, may ultimately affect C storage and nutrient retention, which are also important ecosystem services provided by seagrasses (Nielsen et al., 2004; Fourqurean et al., 2012), as C storage plays a key role in mitigating anthropogenic CO_2_ emissions under the current climate change scenario. The biochemical disproportion caused by these stressors might eventually cause a biomass imbalance, increasing the AG:BG biomass ratios that will reduce the sediment stabilization or oxygenation capacity (Pedersen et al., 1998; Christianen et al., 2013), which are essential ecosystem services provided by seagrass meadows. Moreover, internal C reserves, as sugar and starch concentrations in the rhizome, are important energy suppliers under stressful events such as physical perturbations, shading or nutrient limitation (Soissons et al., 2018). Therefore, the diminishing C storage products would also be expected to exacerbate seagrass decline and to depress the ability of plants to survive dehiscence and dormancy periods (Burkholder et al., 2007). In fact, *T. hemprichii*, and not *C. serrulata*, was present in a highly eutrophic seagrass meadow in the Stone Town area (Zanzibar Archipelago, Tanzania), confirming that the former species have higher tolerance to these conditions even under long-term natural conditions (Teichberg et al., *in preparation*). Similarly, *T. hemprichii* was pointed as the most persistent species in other highly impacted seagrass meadows in China (Thomsen et al., 2020). Therefore, the unequal effect of eutrophication in different species might cause a biodiversity loss in tropical seagrass meadows.

Enhanced leaf morphological traits, primarily observed in *T. hemprichii*, may limit the biomechanical properties and the exposed surface area of the blades, which directly affect survival under high water dynamics during storms (La Nafie et al., 2012). *H. stipulacea*, contrary to the large blades species, did not appear to be negatively affected by the studied factors, showing potentially a dormancy strategy under the different treatments. Even though performance differences have been observed between individuals from its native and invasive ranges (Nguyen et al., 2020; Wesselmann et al., 2020) this species is generally recognized to be tolerant to a wide range of trophic conditions and temperatures (Winters et al., 2020). Although *H. stipulacea* is considered as invasive in the Mediterranean and Caribbean Seas, there is no evidence of competition and displacement of native species in these areas (Boudouresque et al., 2009; Al-Rousan et al., 2011; Gambi et al., 2018; Apostolaki et al., 2019). In addition, the provision of essential ecosystems services by this little seagrass is still under debate and needs further research (Apostolaki et al., 2019; Viana et al., 2019b; Muthukrishnan et al., 2020).

As the duration and frequency of heat waves has increased worldwide in the last century and is expected to continue increasing (Oliver et al., 2018), *C. serrulata* may show some advantages over *T. hemprichii* under these increasing temperature scenarios, at least under the target temperature of this study. On the other side, *T. hemprichii* may perform better under nutrient over-enrichment scenarios, even at higher temperature. If conditions are too detrimental for these species to grow, in terms of light deprivation or anoxic conditions in the sediment, *H. stipulacea* could potentially colonize the area, as it has been shown to be the most plastic and tolerant species in this study and, in general, among other seagrass species.

Overall, the tropical seagrasses in this study showed different tolerances and strategies to cope with stressors, but these responses could be critical in the long run for the seagrass survival and meadow persistence, compromising the maintenance of functions and services provided by the seagrass meadows. Seagrass individual trait responses to stressors are important as a first step to understand the upscaling of ecological consequences of climate change and eutrophication on ecosystem functioning and services. How the individual responses affect their functioning and their abiotic and biotic interactions, however, is an issue that cannot be answered with this experiment and needs further study.

## Conclusion

The holistic picture of seagrass responses highlights that the acclimation and resistance mechanisms behind photosynthesis are closely related with the whole-plant physiology, affecting the performance of the species in the long run. Seagrass species sharing the same original geographic area, or even the same meadow, may respond differently to temperature and nutrient conditions due to unique life history traits. *T. hemprichii* was positively influenced by nutrient over-enrichment conditions, while *H. stipulacea* was tolerant to these conditions, and *C. serrulata* was negatively affected. On the contrary, *C. serrulata* showed a better acclimation under HT scenarios than *H. stipulacea* and *T. hemprichii*. Interaction of both factors negatively affected some important traits of the three species.

Therefore, different scenarios might show winners and losers, but there is no trait that makes a winner under all circumstances, suggesting that if conditions change, some species survivorship might be endangered. Tropical seagrass meadows are characterized by high species diversity, but their functional roles are not always clearly interchangeable, as within the species in this study, and displacement of any of the three species may cause a functional or service loss of the seagrass community as a whole (Duarte, 2000).

## Data Availability Statement

The original contributions presented in the study are included in the article/Supplementary Material. Further inquiries can be directed to the corresponding author.

## Author Contributions

MT got the project funding. MT and IV designed the experiment. AM-S and IV carried out the experiment and processed the samples. AM-S and IV analyzed morphological, growth and nutrient data. AM-S analyzed water and PAM data. IV wrote a first version of the manuscript. MT and AM-S made significant contributions to the manuscript and critically revised the different versions of the manuscript. All authors contributed to the article and approved the submitted version.

## Conflict of Interest

The authors declare that the research was conducted in the absence of any commercial or financial relationships that could be construed as a potential conflict of interest.
