# Supplementary material for: Species-Specific Trait Responses of Three Tropical Seagrasses to Multiple Stressors: The Case of Increasing Temperature and Nutrient Enrichment
Source: Front Plant Sci. 2020 Nov 5;11:571363. doi: 10.3389/fpls.2020.571363 (PMC7674176; doi:10.3389/fpls.2020.571363)
Supplement: Supplementary file 1 [file Data_Sheet_1.docx]

**
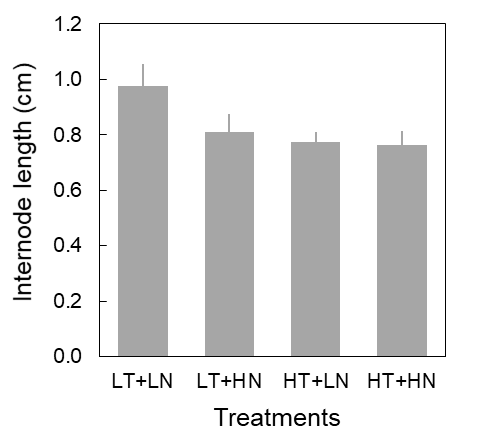
**

**Supplementary Figure 1.** *Halophila stipulacea* internode length (mean±SE, n=6) in the four treatments (LT: low temperature; LN: low nutrient; HT: high temperature; HN: high nutrient). Statistical results are shown in **Table 3**.

**Supplementary Table 1.** Free amino acids concentrations in the four treatments (LT: low temperature; LN: low nutrient; HT: high temperature; HN: high nutrient).

|  | *T. hemprichii* | | | |  | *C. serrulata* | | | |
| --- | --- | --- | --- | --- | --- | --- | --- | --- | --- |
|  | LT+LN | LT+HN | HT+LN | HT+HN |  | LT+LN | LT+HN | HT+LN | HT+HN |
| ALA | 0.38±0.07 | 0.33±0.04 | 0.43±0.07 | 0.35±0.05 |  | 0.39±0.07 | 0.48±0.06 | 0.28±0.04 | 0.39±0.07 |
| ARG | 0.05±0.004 | 0.05±0.001 | 0.06±0.002 | 0.05±0.005 |  | 0.07±0.004 | 0.07±0.004 | 0.06±0.002 | 0.07±0.004 |
| ASP | 1.03±0.15 | 1.19±0.14 | 1.16±0.18 | 0.99±0.2 |  | 0.77±0.1 | 0.81±0.08 | 0.54±0.07 | 0.77±0.1 |
| GLU | 0.78±0.14 | 0.85±0.1 | 0.74±0.07 | 0.63±0.08 |  | 0.71±0.12 | 0.85±0.13 | 0.56±0.06 | 0.86±0.12 |
| GLY | 0.10±0.01 | 0.11±0.01 | 0.13±0.01 | 0.09±0.01 |  | 0.18±0.02 | 0.21±0.03 | 0.17±0.01 | 0.17±0.02 |
| HIS | 0.13±0.01 | 0.11±0.01 | 0.12±0.01 | 0.11±0.01 |  | 0.13±0.02 | 0.15±0.01 | 0.16±0.02 | 0.17±0.02 |
| ILE | 0.09±0.01 | 0.08±0.01 | 0.1±0.01 | 0.07±0.005 |  | 0.12±0.02 | 0.13±0.02 | 0.14±0.01 | 0.13±0.02 |
| LEU | 0.08±0.01 | 0.07±0.002 | 0.08±0.004 | 0.07±0.001 |  | 0.1±0.01 | 0.13±0.02 | 0.12±0.02 | 0.1±0.01 |
| MET | 0.06±0.005 | 0.06±0.002 | 0.06±0.003 | 0.05±0.004 |  | 0.06±0.003 | 0.06±0.003 | 0.05±0.01 | 0.06±0.003 |
| PHE | 0.11±0.01 | 0.10±0.01 | 0.11±0.01 | 0.09±0.005 |  | 0.12±0.01 | 0.16±0.03 | 0.14±0.01 | 0.14±0.01 |
| THR | 0.82±0.09 | 0.74±0.29 | 0.39±0.1 | 0.43±0.09 |  | 0.53±0.13 | 0.88±0.27 | 0.41±0.08 | 0.43±0.13 |
| TYR | 0.06±0.005 | 0.05±0.002 | 0.06±0.003 | 0.05±0.002 |  | 0.06±0.004 | 0.08±0.01 | 0.07±0.005 | 0.06±0.004 |
| SER | 0.35±0.06 | 0.29±0.04 | 0.32±0.03 | 0.23±0.03 |  | 0.47±0.1 | 0.47±0.05 | 0.48±0.08 | 0.56±0.1 |
| VAL | 0.13±0.02 | 0.10±0.01 | 0.12±0.01 | 0.1±0.01 |  | 0.16±0.02 | 0.19±0.03 | 0.17±0.01 | 0.16±0.02 |

**Supplementary Table 2.** *Thalassia hemprichii* Pearson correlation coefficients (lower semimatrix) and significance (upper semimatrix) between biochemical, morphological and physiological traits. Green cells indicate significantly positive correlations while red cells indicate significantly inverse correlations (*P-value*: *: <0.05, **: ≤0.01, ***: ≤0.001). Values in bold represent statistical significance at the 10% level of significance (FAA: free amino acids).

|  | Leaf C:N | Leaf N | Leaf C | Leaf FAA | Rhizome N | Rhizome C | Sucrose | Starch | Root N | Root C | Leaf length | Leaf SA | Sheath length | Root length | Growth | F_v_/F_m_ | rETR_max_ | Alpha | E_k_ |
| --- | --- | --- | --- | --- | --- | --- | --- | --- | --- | --- | --- | --- | --- | --- | --- | --- | --- | --- | --- |
| Leaf C:N | - | <0.001 | <0.001 | 0.34 | 0.75 | 0.32 | 0.75 | 0.87 | 0.95 | 0.88 | 0.60 | 0.81 | 0.25 | 0.15 | 0.20 | 0.30 | 0.49 | 0.17 | 0.36 |
| Leaf N | -0.97^***^ | - | <0.001 | 0.37 | 0.95 | 0.19 | 0.75 | 0.57 | 0.94 | 0.86 | 0.50 | 0.91 | 0.25 | 0.23 | 0.11 | 0.23 | 0.26 | 0.21 | 0.59 |
| Leaf C | -0.68^***^ | 0.81^***^ | - | 0.38 | 0.55 | 0.09 | 0.82 | 0.24 | 0.52 | 0.61 | 0.27 | 0.85 | 0.29 | 0.75 | **0.08** | 0.44 | 0.03 | 0.90 | 0.51 |
| Leaf FAA | -0.20 | 0.19 | 0.19 | - | 0.61 | 0.42 | 0.42 | 0.38 | 0.02 | 0.02 | 0.31 | 0.37 | 0.13 | 0.83 | 0.04 | 0.63 | 0.03 | 0.27 | 0.05 |
| Rhizome N | 0.07 | -0.01 | 0.13 | 0.11 | - | **0.08** | **0.06** | 0.14 | 0.005 | 0.03 | 0.39 | 0.99 | 0.38 | 0.05 | 0.47 | 0.22 | 0.46 | 0.45 | 0.96 |
| Rhizome C | 0.21 | -0.28 | -0.35 | 0.17 | **0.36** | - | 0.68 | 0.02 | 0.04 | 0.03 | 0.22 | 0.20 | 0.33 | 0.56 | 0.86 | 0.95 | 0.74 | 0.95 | 0.95 |
| Sucrose | 0.07 | -0.07 | -0.05 | -0.17 | **-0.40** | -0.09 | - | 0.54 | 0.13 | 0.02 | 0.49 | 0.73 | 0.35 | **0.08** | 0.81 | 0.27 | 0.24 | 0.03 | **0.08** |
| Starch | 0.03 | -0.12 | -0.25 | 0.19 | -0.31 | 0.46^*^ | 0.13 | - | 0.52 | 0.33 | 0.10 | 0.13 | 0.09 | 0.10 | 0.39 | 0.60 | 0.45 | 0.48 | 0.56 |
| Root N | 0.01 | 0.02 | 0.14 | 0.47^*^ | 0.55^**^ | 0.42^*^ | -0.32 | 0.14 | - | <0.001 | 0.53 | 0.39 | 0.48 | 0.34 | 0.12 | 0.82 | 0.02 | 0.90 | 0.32 |
| Root C | -0.03 | 0.04 | 0.11 | 0.48^*^ | 0.44^*^ | 0.45^*^ | -0.48^*^ | 0.21 | 0.93^***^ | - | 0.45 | 0.21 | 0.52 | 0.35 | 0.15 | 0.73 | 0.16 | 0.73 | 0.99 |
| Leaf length | -0.11 | 0.14 | 0.24 | -0.22 | 0.19 | -0.26 | -0.15 | -0.34 | -0.13 | -0.16 | - | 0.001 | <0.001 | 0.30 | 0.14 | 0.22 | 0.75 | 0.82 | 0.87 |
| Leaf SA | 0.05 | -0.02 | -0.04 | -0.19 | 0.00 | -0.27 | -0.07 | -0.31 | -0.18 | -0.27 | 0.63^***^ | - | 0.01 | 0.54 | 0.62 | 0.62 | 0.43 | 0.86 | 0.67 |
| Sheath length | -0.24 | 0.24 | 0.23 | -0.32 | 0.19 | -0.21 | -0.20 | -0.35 | -0.15 | -0.14 | 0.92^***^ | 0.51^*^ | - | 0.23 | **0.06** | 0.17 | 0.58 | 0.36 | 0.54 |
| Root length | -0.30 | 0.25 | 0.07 | 0.05 | 0.40* | -0.13 | **-0.37** | -0.34 | 0.20 | 0.20 | 0.22 | 0.13 | 0.26 | - | 0.84 | 0.78 | 0.07 | 0.26 | 0.15 |
| Growth | -0.27 | 0.34 | **0.37** | -0.43^*^ | 0.15 | -0.04 | 0.05 | -0.18 | -0.33 | -0.31 | 0.31 | -0.11 | **0.40** | 0.04 | - | 0.31 | 0.52 | 0.32 | 0.28 |
| F_v_/F_m_ | -0.22 | 0.25 | 0.16 | -0.10 | 0.26 | 0.01 | -0.24 | -0.11 | -0.05 | -0.08 | 0.26 | 0.11 | 0.29 | -0.06 | 0.22 | - | 0.53 | <0.001 | 0.74 |
| rETR_max_ | -0.15 | 0.24 | 0.44^*^ | 0.44^*^ | 0.16 | 0.07 | 0.25 | 0.16 | 0.46^*^ | 0.30 | -0.07 | -0.17 | -0.12 | -0.38 | -0.14 | 0.14 | - | 0.36 | 0.002 |
| Alpha | -0.29 | 0.26 | 0.03 | -0.24 | 0.16 | -0.01 | -0.45^*^ | -0.15 | -0.03 | 0.07 | 0.05 | 0.04 | 0.19 | 0.24 | 0.21 | 0.73^**^ | -0.20 | - | 0.001 |
| E_k_ | 0.19 | -0.11 | 0.14 | 0.41^*^ | 0.01 | 0.01 | **0.36** | 0.12 | 0.21 | 0.00 | 0.03 | -0.09 | -0.13 | -0.31 | -0.23 | -0.07 | 0.61^**^ | -0.64^***^ | - |

**Supplementary Table 3.** *Cymodocea serrulata* Pearson correlation coefficients (lower semimatrix) and significance (upper semimatrix) between biochemical, morphological and physiological traits. Green cells indicate significantly positive correlations while red cells indicate significantly inverse correlations (*P-value*: *: <0.05, **: ≤0.01, ***: ≤0.001). Values in bold represent statistical significance at the 10% level of significance (FAA: free amino acids).

|  | Leaf C:N | Leaf N | Leaf C | Leaf FAA | Rhizome N | Rhizome C | Sucrose | Starch | Root N | Root C | Leaf length | Leaf SA | Sheath length | Root length | Growth | F_v_/F_m_ | rETR_max_ | Alpha | E_k_ |
| --- | --- | --- | --- | --- | --- | --- | --- | --- | --- | --- | --- | --- | --- | --- | --- | --- | --- | --- | --- |
| Leaf C:N | - | <0.001 | 0.05 | 0.56 | 0.35 | 0.24 | 0.81 | **0.06** | 0.01 | 0.003 | 0.12 | **0.08** | 0.13 | 0.57 | 0.19 | 0.62 | 0.42 | 0.41 | 0.64 |
| Leaf N | -0.93^***^ | - | 0.01 | 0.76 | 0.76 | 0.12 | 0.45 | 0.02 | 0.03 | 0.003 | **0.07** | 0.04 | 0.04 | 0.25 | 0.17 | 0.71 | 0.34 | 0.31 | 0.50 |
| Leaf C | -0.41* | 0.52^**^ | - | 0.75 | 0.52 | 0.88 | 0.45 | 0.45 | 0.10 | **0.08** | <0.001 | <0.001 | 0.01 | 0.33 | 0.83 | 0.41 | 0.72 | 0.24 | 0.58 |
| Leaf FAA | -0.13 | 0.06 | 0.07 | - | 0.42 | 0.83 | 0.93 | 0.36 | 0.11 | 0.09 | 0.10 | 0.10 | 0.39 | 0.04 | 0.74 | 0.45 | 0.75 | 0.75 | 0.59 |
| Rhizome N | 0.20 | -0.07 | 0.14 | 0.17 | - | 0.91 | 0.96 | 0.81 | 0.04 | 0.25 | 0.21 | 0.24 | 0.14 | 0.89 | 0.98 | 0.37 | 0.27 | 0.58 | 0.48 |
| Rhizome C | 0.25 | -0.33 | 0.03 | 0.04 | 0.02 | - | 0.49 | 0.01 | 0.78 | **0.08** | 0.86 | 1.00 | 0.28 | 1.00 | 0.06 | 0.49 | 0.02 | 0.40 | 0.01 |
| Sucrose | 0.05 | -0.17 | -0.17 | 0.02 | 0.01 | 0.15 | - |  | 0.11 | 0.01 | 0.89 | 0.90 | 0.43 | 0.48 | 0.18 | 0.88 | 0.22 | 0.84 | 0.27 |
| Starch | **0.39** | -0.47^*^ | -0.17 | -0.20 | -0.05 | 0.55^**^ | 0.17 | - | 0.63 | 0.35 | 0.25 | 0.39 | 0.36 | 0.61 | 0.17 | 0.35 | 0.01 | 0.69 | 0.01 |
| Root N | 0.49^*^ | -0.45^*^ | -0.36 | -0.33 | 0.42^*^ | 0.06 | 0.34 | 0.10 | - | <0.001 | 0.48 | 0.29 | 0.64 | 1.00 | 0.59 | 0.73 | 0.83 | 0.77 | 0.66 |
| Root C | 0.59^**^ | -0.57^**^ | **-0.38** | -0.35 | 0.24 | **0.36** | 0.52^*^ | 0.20 | 0.82^**^ | - | 0.27 | 0.29 | 0.34 | 0.77 | 0.58 | 0.89 | 0.59 | 0.71 | 0.83 |
| Leaf length | 0.33 | **-0.38** | -0.71^***^ | -0.35 | -0.27 | 0.04 | 0.03 | 0.25 | 0.15 | 0.23 | - | <0.001 | <0.001 | 0.53 | 0.71 | **0.08** | 0.18 | 0.03 | 0.11 |
| Leaf SA | **0.36** | -0.42^*^ | -0.73^***^ | -0.34 | -0.25 | 0.00 | -0.03 | 0.18 | 0.23 | 0.23 | 0.95^***^ | - | <0.001 | 0.46 | 0.84 | 0.03 | 0.12 | 0.004 | **0.08** |
| Sheath length | 0.32 | -0.43^*^ | -0.55^**^ | -0.19 | -0.31 | 0.23 | 0.17 | 0.20 | -0.10 | 0.20 | 0.82^***^ | 0.78^**^ | - | 0.26 | 0.68 | **0.06** | 0.58 | 0.05 | 0.69 |
| Root length | 0.12 | -0.25 | -0.21 | 0.42^*^ | -0.03 | 0.00 | -0.16 | -0.11 | 0.00 | -0.06 | 0.14 | 0.16 | 0.24 | - | 0.50 | 0.47 | 0.92 | 0.37 | 0.78 |
| Growth | -0.28 | 0.29 | 0.05 | -0.07 | 0.00 | -0.38 | -0.29 | -0.29 | -0.11 | -0.12 | 0.08 | 0.04 | -0.09 | -0.14 |  | 0.43 | 0.01 | 0.12 | 0.002 |
| F_v_/F_m_ | 0.11 | -0.08 | -0.18 | 0.16 | 0.19 | -0.15 | 0.03 | -0.20 | -0.07 | -0.03 | **0.37** | 0.45^*^ | **0.40** | -0.15 | 0.17 | - | 0.03 | <0.001 | **0.07** |
| rETR_max_ | 0.17 | -0.21 | 0.08 | 0.07 | 0.24 | 0.46^*^ | 0.26 | 0.53^**^ | 0.05 | 0.11 | -0.28 | -0.33 | -0.12 | -0.02 | -0.52^**^ | -0.45^*^ | - | <0.001 | <0.001 |
| Alpha | 0.17 | -0.22 | -0.26 | -0.07 | -0.12 | -0.18 | 0.04 | -0.09 | 0.06 | 0.08 | 0.45^*^ | 0.57^**^ | 0.40* | -0.19 | 0.33 | 0.80^***^ | -0.62^**^ | - | 0.01 |
| E_k_ | 0.10 | -0.14 | 0.12 | 0.12 | 0.15 | 0.54^**^ | 0.24 | 0.53^**^ | -0.09 | 0.05 | -0.33 | **-0.37** | -0.09 | -0.06 | -0.60^**^ | **-0.37** | 0.90^**^ | -0.55^**^ | - |

**Supplementary Table 4.** *Halophila stipulacea* Pearson correlation coefficients (lower semimatrix) and significance (upper semimatrix) between biochemical, morphological and physiological traits. Green cells indicate significantly positive correlations while red cells indicate significantly inverse correlations (*P-value*: *: <0.05, **: ≤0.01, ***: ≤0.001). Values in bold represent statistical significance at the 10% level of significance (AG: above-ground tissues, BG: below-ground tissues, SA: surface area, IL: internode length).

|  | AG C:N | AG N | AG C | BG N | BG C | Sucrose | Starch | Leaf length | Leaf SA | Petiole length | Root length | IL | F_v_/F_m_ | rETR_max_ | Alpha | E_k_ |
| --- | --- | --- | --- | --- | --- | --- | --- | --- | --- | --- | --- | --- | --- | --- | --- | --- |
| AG C:N | - | <0.001 | 0.52 | 0.35 | 0.88 | 0.18 | 0.75 | 0.23 | 0.68 | 0.12 | 0.72 | 0.59 | 0.31 | 0.25 | 0.92 | 0.16 |
| AG N | -0.90^***^ | - | 0.04 | 0.41 | 0.73 | 0.18 | 0.37 | 0.39 | 0.81 | **0.07** | 0.97 | 0.69 | 0.15 | 0.38 | 0.54 | 0.34 |
| AG C | -0.21 | 0.61^*^ | - | 0.87 | 0.26 | 0.54 | **0.08** | 0.81 | 0.85 | 0.24 | 0.48 | 0.87 | 0.11 | 0.99 | 0.12 | 0.79 |
| BG N | 0.30 | -0.26 | -0.05 | - | **0.08** | 0.90 | 0.91 | 0.59 | 0.54 | 0.52 | 0.60 | 0.36 | 0.81 | 0.58 | 0.89 | 0.69 |
| BG C | -0.05 | -0.11 | -0.35 | **0.52** | - | 0.13 | 0.21 | 0.05 | 0.16 | 0.89 | 0.03 | 0.83 | 0.94 | 0.11 | 0.33 | 0.09 |
| Sucrose | 0.44 | -0.44 | -0.21 | 0.04 | 0.48 | - | 0.48 | 0.04 | **0.07** | 0.42 | 0.04 | 0.41 | 0.33 | 0.69 | 0.32 | 0.63 |
| Starch | 0.11 | -0.30 | **-0.55** | 0.04 | 0.41 | 0.24 | - | 0.63 | 0.85 | 0.17 | 0.23 | 0.96 | 0.45 | 0.47 | 0.16 | 0.19 |
| Leaf length | -0.37 | 0.27 | -0.08 | -0.18 | 0.58^*^ | 0.62^*^ | 0.16 | - | 0.002 | 0.02 | 0.004 | 0.19 | 0.92 | 0.39 | 0.55 | 0.15 |
| Leaf SA | -0.13 | 0.08 | -0.06 | -0.20 | 0.43 | **0.56** | 0.06 | 0.80^**^ | - | 0.06 | 0.001 | 0.05 | 0.89 | 0.43 | 0.62 | 0.19 |
| Petiole length | -0.48 | **0.54** | 0.37 | -0.21 | 0.05 | 0.27 | -0.44 | 0.67^*^ | 0.57 | - | 0.21 | 0.01 | 0.42 | 0.62 | 0.66 | 0.50 |
| Root length | -0.12 | -0.01 | -0.23 | -0.17 | 0.61^*^ | 0.62^*^ | 0.39 | 0.77^**^ | 0.83^***^ | 0.39 | - | 0.02 | 0.95 | **0.07** | 0.41 | 0.004 |
| IL | -0.17 | 0.13 | -0.05 | -0.29 | 0.07 | 0.28 | 0.02 | 0.28 | 0.40* | 0.53^**^ | 0.49* | - | 0.78 | 0.34 | 0.43 | **0.07** |
| F_v_/F_m_ | -0.32 | 0.44 | 0.49 | 0.08 | -0.03 | -0.32 | -0.26 | 0.03 | -0.04 | 0.26 | 0.02 | -0.09 | - | **0.07** | <0.001 | 0.64 |
| rETR_max_ | -0.36 | 0.28 | 0.01 | 0.18 | 0.48 | -0.14 | 0.25 | 0.27 | 0.25 | 0.16 | **0.54** | 0.30 | **0.53** | - | 0.67 | 0.01 |
| Alpha | -0.03 | 0.20 | 0.48 | 0.04 | -0.31 | -0.33 | -0.46 | -0.19 | -0.16 | 0.14 | -0.26 | -0.25 | 0.88^***^ | 0.14 | - | 0.36 |
| E_k_ | -0.43 | 0.30 | -0.09 | -0.13 | 0.50 | 0.17 | 0.43 | 0.45 | 0.40 | 0.21 | 0.77^**^ | **0.54** | 0.15 | 0.74^**^ | -0.29 | - |
